# Assessing the Association between Natural Food Folate Intake and Blood Folate Concentrations: A Systematic Review and Bayesian Meta-Analysis of Trials and Observational Studies

**DOI:** 10.3390/nu7042663

**Published:** 2015-04-10

**Authors:** Claire M. Marchetta, Owen J. Devine, Krista S. Crider, Becky L. Tsang, Amy M. Cordero, Yan Ping Qi, Jing Guo, Robert J. Berry, Jorge Rosenthal, Joseph Mulinare, Patricia Mersereau, Heather C. Hamner

**Affiliations:** 1 Oak Ridge Institute for Science and Education (ORISE), Oak Ridge, TN 37831, USA; E-Mails: claire.marchetta@gmail.com (C.M.M.); bltsang@gmail.com (B.L.T.); rv7@cdc.gov (Y.P.Q.); 2 Carter Consulting, Inc., Atlanta, GA 30345, USA; E-Mails: ojd1@cdc.gov (O.J.D.); jxm1@cdc.gov (J.M.); 3 Division of Birth Defects and Developmental Disabilities (DBDDD), National Center on Birth Defects and Developmental Disabilities (NCBDDD), Centers for Disease Control and Prevention, Atlanta, GA 30329, USA; E-Mails: kvc3@cdc.gov (K.S.C.); iqt8@cdc.gov (A.M.C.); rjb1@cdc.gov (R.J.B.); jyr4@cdc.gov (J.R.); 4 Acentia, Falls Church, VA 22042, USA; E-Mail: shashagj@gmail.com; 5 SciMetrika, LLC, Atlanta, GA 30329, USA; E-Mail: pmersereau@comcast.net; 6 Division of Nutrition, Physical Activity, and Obesity (DNPAO), National Center for Chronic Disease Prevention and Health Promotion (NCCDPHP), Centers for Disease Control and Prevention, Atlanta, GA 30329, USA

**Keywords:** food folate, serum/plasma folate, RBC folate

## Abstract

Folate is found naturally in foods or as synthetic folic acid in dietary supplements and fortified foods. Adequate periconceptional folic acid intake can prevent neural tube defects. Folate intake impacts blood folate concentration; however, the dose-response between natural food folate and blood folate concentrations has not been well described. We estimated this association among healthy females. A systematic literature review identified studies (1 1992–3 2014) with both natural food folate intake alone and blood folate concentration among females aged 12–49 years. Bayesian methods were used to estimate regression model parameters describing the association between natural food folate intake and subsequent blood folate concentration. Seven controlled trials and 29 observational studies met the inclusion criteria. For the six studies using microbiologic assay (MA) included in the meta-analysis, we estimate that a 6% (95% Credible Interval (CrI): 4%, 9%) increase in red blood cell (RBC) folate concentration and a 7% (95% CrI: 1%, 12%) increase in serum/plasma folate concentration can occur for every 10% increase in natural food folate intake. Using modeled results, we estimate that a natural food folate intake of ≥450 μg dietary folate equivalents (DFE)/day could achieve the lower bound of an RBC folate concentration (~1050 nmol/L) associated with the lowest risk of a neural tube defect. Natural food folate intake affects blood folate concentration and adequate intakes could help women achieve a RBC folate concentration associated with a risk of 6 neural tube defects/10,000 live births.

## 1. Introduction

Folate, the umbrella term used to describe both natural food folate and synthetic folic acid, is necessary for basic cellular functions. Natural food folate can be found in liver, dark green leafy vegetables, legumes, and some fruits, such as oranges [1]. Natural food folate’s bioavailability is less than synthetic folic acid [1,2,3] in part because it must undergo deconjugation before it can be absorbed and made available for metabolic reactions and/or storage. Research has indicated that relative to the consumption of folic acid with food, the bioavailability of natural food folate is approximately 50% [2]. The difference in bioavailability can be described using the following equation: 1 μg Dietary Folate Equivalents (DFE) = 0.6 μg folic acid [2]. DFEs are a unit of measurement that reflect the greater bioavailability of folic acid compared to natural food folate [2].

Folate status can be assessed using either serum/plasma folate (short-term indicator) or red blood cell (RBC) folate (long-term indicator) concentrations [1,2,3]. Folate deficiency, defined by the World Health Organization (WHO) as serum/plasma folate concentrations <10 nmol/L or RBC folate concentrations <340 nmol/L, is based on elevated circulating homocysteine concentrations [4]. However, it has been shown that folate-preventable birth defects of the brain and spine (neural tube defects) can occur at blood folate concentrations above the defined deficiency cut-offs [5]. For the “optimal” prevention of neural tube defect-affected pregnancies among women of childbearing age, RBC folate concentrations of ≥906 nmol/L [5] and ≥ about 1000 nmol/L [6] have been suggested.

Although research has shown that folic acid intake increases blood folate concentrations [7,8] and periconceptional folic acid supplementation and folic acid fortification of staple foods reduces the risk of a neural tube defect-affected pregnancy [9,10,11,12,13], there are limited data available on the unique contribution of natural food folate intake to blood folate concentrations and the potential to reach “optimal” blood folate concentrations to prevent neural tube defects through natural food folate intake alone. A better understanding of the associated dose-response between natural food folate intake and blood folate concentrations could improve the development, monitoring, and evaluation of neural tube defect prevention programs. This is of particular interest in settings where periconceptional folic acid supplement use is low or folic acid fortified staple foods are not available.

Thus, the objective of our analysis was to determine the association and estimate the dose-response between natural food folate intake and blood folate concentration using a meta-analysis of studies identified through a systematic literature review.

## 2. Subjects and Methods

Many of the methods, including the search strategy, screening criteria, and quality assessment methods, for this review are shared with a separate systematic review conducted by our review team, and have been described previously [14]; however, these methods are detailed below and in the protocol developed by all coauthors (Supplementary S1). We adhered to guidelines from the Cochrane Handbook for Systematic Reviews [15] and the Preferred Reporting Items for Systematic Reviews and Meta-Analyses (PRISMA) statement [16]. The methods below are summarized in brief.

### 2.1. Search Strategy

A research librarian from CDC’s Public Health Library and Information Center conducted a search for English language studies published between 1 January 1992 and 7 March 2014. The year 1992 was set as a limit because it corresponds to the US Public Health Service recommendation for all women of childbearing age to consume 400 μg of folic acid daily for the prevention of neural tube defects [10]. The following databases were searched: PubMed (includes Medline), Embase, Cumulative Index to Nursing and Allied Health (CINAHL), Cochrane Library, Web of Science, and Population Information Online (POPLINE). The search strategy for Embase included keywords in the following areas: Folic acid, blood folate (blood folate or folic acid blood level, serum folate or plasma folate or red blood cell folate), intake (intake or diet * or supplement *, folic acid intake), and women of childbearing age (childbear * or women * or female * or girl * or pregnant *). This method was adapted for the other databases searched. We also hand-searched reference lists from articles selected for abstraction for additional relevant citations not captured in the database searches.

### 2.2. Inclusion and Exclusion Criteria

Population eligibility criteria were nonpregnant, nonlactating females aged 12–49 years who had not consumed folic acid containing supplements or folic acid fortified foods during the period of dietary intake assessment. If pregnancy/lactation status was not explicitly stated for the studied population, we assumed the participants were not pregnant or lactating. Required reported data were blood folate data (serum, plasma, or RBC folate concentration), blood folate assay methodology, and natural food folate intake amount.

We contacted authors for stratified data if populations were a mix of males and females, had participants outside the 12–49 target age range, indicated inclusion of pregnant/lactating women, and/or included participants who had consumed folic acid containing supplements or fortified foods. Studies (unique articles/publications) were excluded if they did not meet the inclusion criteria, targeted an unhealthy population, or if stratified data were not available due to author nonresponse. Subsequently, studies were classified as either Tier 1 or Tier 2; Tier 1 studies pertained to data for nonpregnant, nonlactating females 12–49 years of age only. Tier 2 studies were classified as such if age/sex stratified data were unavailable but more than half of the participants (at least 51%) were female and the mean or median female age was between 12 and 49 years. Tier 2 studies were not included in the meta-analysis.

### 2.3. Selection of Articles

**Figure 1:**
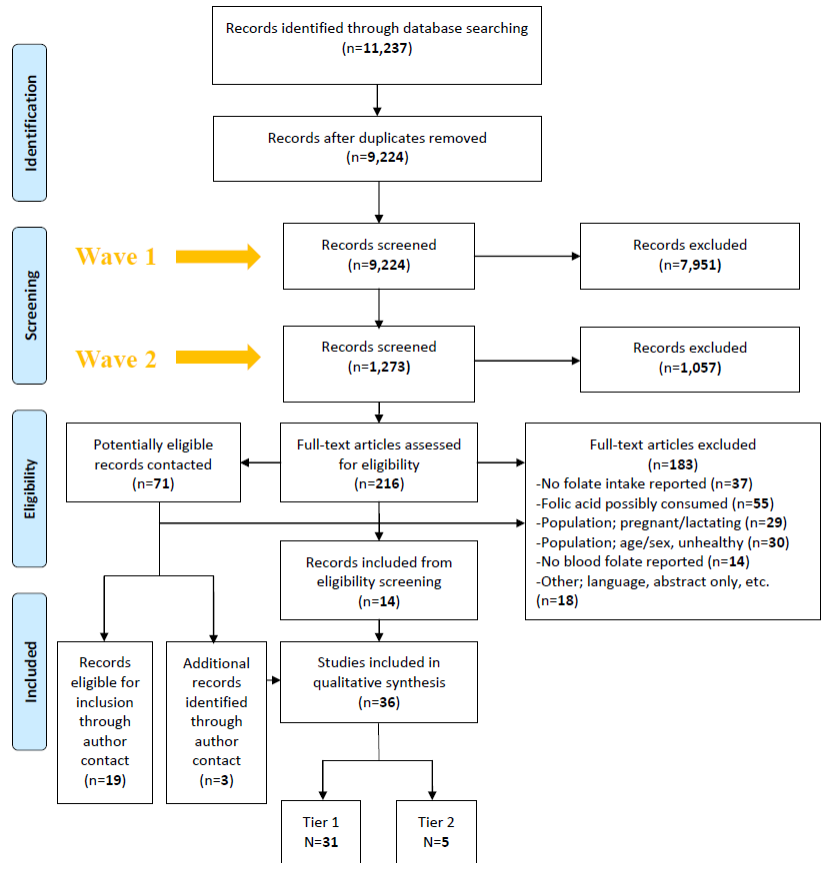
Record management and selection flow diagram for the systematic review on natural food folate intake and blood folate concentrations. Wave 1 screening was a review of abstracts with a more broad focus. Wave 2 screening was a review of abstracts using the specific objectives from this study. Full text articles excluded (*n* = 183) is the sum of the 131 articles initially rejected after the full text review plus the 52 articles rejected after contacting authors. The final number of studies included in the qualitative synthesis (*n* = 36) is the sum of records accepted from full-text eligibility screening (*n* = 14), records eligible for inclusion through author contact (*n* = 19) and additional records identified through author contact (*n* =3).

Abstract and title review consisted of a “Wave 1” and “Wave 2” screening (Figure 1). In Wave 1, three teams of two reviewers (Claire M. Marchetta + Jorge Rosenthal; Robert J. Berry + Heather C. Hamner; Patricia Mersereau + Joe Mulinare) independently screened a third of the titles and abstracts for inclusion, using criteria relevant for this review as well as the aforementioned Tsang *et al.* review [14]. In Wave 2, inclusion/exclusion criteria specific to this review were applied independently by three reviewers (Claire M. Marchetta, Jorge Rosenthal, Robert J. Berry). Full text review was conducted by the same three reviewers. Reviewers resolved any disagreement regarding inclusion/exclusion by discussion. Two attempts were made to contact authors for additional information.

### 2.4. Data Extraction

Three reviewers (Claire M. Marchetta, Jorge Rosenthal, Robert J. Berry) each took a third of all identified studies and used a prepiloted abstraction form to extract data on study design, intervention (if applicable), selection of population, natural food folate intake, dietary measurement method, blood folate concentrations at baseline and follow-up (if applicable), and blood folate assay method. Other extracted information included study location, sample size, study years, country fortification status, and participant characteristics (age and ethnicity). A fourth investigator (Heather C. Hamner) reviewed all studies and abstracted data to check for accuracy and consistency.

### 2.5. Quality Assessment of Studies

Risk of bias assessment methods in detail have been previously described [14]. In brief, a risk of bias assessment was conducted separately according to outcome (RBC folate and/or serum/plasma folate) for all studies included in the systematic review using one of two methods. For controlled trials, the risk of bias was assessed using the *Cochrane Handbook for Systematic Reviews of Interventions* tool [15]. Cohort and cross-sectional studies were assessed using the Item Bank on Risk of Bias and Precision of Observational Studies from RTI International (Table S1) [17]. Both tools were adapted to the objectives of this review and piloted before use. Two researchers who had not participated in study selection independently conducted the risk of bias assessment in duplicate (Yan Ping Qi, Jing Guo). Any disagreements were resolved by discussion.

### 2.6. Data Standardization

Natural food folate intake measurements and blood folate concentrations were standardized prior to meta-analysis because the information extracted from the selected studies was comprised of differing measures of central tendency and variability on differing scales (e.g., means, medians, geometric means, log transformed and untransformed data, standard deviations, 95% confidence intervals, *etc.*). In this analysis, we standardized the reported study results (*i.e.*, natural food folate intake and blood folate concentrations) so that the information extracted from each study corresponded to the mean and the standard error of that mean on the natural log scale for both intake and blood folate concentration. This standardization to matching summary statistics on the log scale was conducted using previously published formulae [18].

To account for documented variations between microbiologic assay (MA) methods [19], blood folate concentrations were adjusted to increase comparability between two different MA methods. To illustrate, let *X* be the MA value derived using the assay method of Tamura [20] and Y be the concentration derived using the assay method of Molloy/O’Broin [21,22]. The association between the MA approaches has been estimated as the following (C. Pfeiffer, Personal communication, 2014):

Serum folate (nmol/L): *Y* = 1.4209 × *X* + 0.7854 RBC folate (nmol/L): *Y* = 0.7297 × *X* + 352.219

We used these models to transform all results to those that would have been derived under the Molloy/O’Broin method. To reflect the uncertainty in this conversion, the sampling variability of the coefficients in the above models were incorporated into the standardized SEMs of the transformed log concentration using Taylor Series approximation [23].

Blood folate concentrations from commercial protein binding assays (PBA) have demonstrated limited inter-assay comparability [24,25]; and because no validated adjustment formulae exist, no attempt was made to standardize blood folate concentrations to account for differing PBA methods across studies.

Although dietary intake assessment methods can vary substantially [26], we did not attempt to standardize these values to a specific method because no validated adjustment formulae were available. It was beyond the scope of our study to ensure that validated questionnaires were used in each study or that dietary analyses were done using specific statistical methodologies.

All data presented in Tables S2 and S3 represent the original study data, with blood folate concentrations presented in nmol/L and the different dietary intake assessment methods noted. Standardized measures of central tendency and standardized blood folate data were used for the meta-analysis only.

### 2.7. Meta-Analysis Study Inclusion/Exclusion

Tier 1 studies were eligible for the meta-analysis; of these, several were excluded. One study [27] was excluded because of implausibly low blood folate data for the reported amount of natural food folate intake (attempts to verify data from the author were unsuccessful). Two studies [28,29] were excluded from the meta-analysis because they reported using a PBA to assess RBC folate concentrations; analyses for RBC folate concentrations were limited to those studies using MA.

Several controlled trial studies were also excluded based on potential exposure to folic acid consumption prior to the study’s initiation. There was potential exposure to folic acid because these studies took place in the United States of America (USA) where there is folic acid fortification of enriched cereal grain products. These studies included depletion/restriction phases; however, these depletion/restriction phase data points were not used if the depletion/restriction phase was less than 120 days (*i.e.*, the lifespan of an RBC) [30,31,32,33]. These folate “restriction” or “depletion” phases ranged from two to seven weeks and were thus too short to reflect true blood folate concentrations at the level of natural food folate intake provided in the study. Given that these studies took place in a setting with mandatory folic acid fortification, without an adequate washout period of at least 120 days, the data were susceptible to contamination by previous folic acid consumption from foods fortified with folic acid or supplements containing folic acid. Therefore, available data from Abratte *et al.*, [30], Perry *et al.* [32], and Shelnutt *et al.* [33] and the restriction data point from Hung *et al.* [31] were not included in the meta-analysis. Intervention time points only from Wright *et al.* and Hung *et al.* [31,34] were used in the meta-analysis. Baseline values from Wright *et al.* [34] were not used to avoid the introduction of potential systematic bias using multiple time points for the same population.

Exploratory analysis indicated that two studies, Kwanbunjan *et al.*, [35] (Supplementary S2: Figures S1 and S3) and Pathak *et al.*, [36] (Supplementary S2: Figure S4) had natural food folate intake and blood folate concentration estimates that were outliers and could be of questionable validity. We were unable to verify the findings from the study authors. Therefore, results from these two studies were omitted from the primary analyses. Sensitivity analyses were conducted to determine the impact on overall interpretations of data (Supplementary S2).

Of the 11 studies that used the MA method, six were included in the meta-analysis, representing a total of nine observations for RBC folate and serum/plasma folate concentrations [31,34,37,38,39,40]. Of the 20 studies that used a PBA method, 17 studies with 22 observations for serum/plasma folate concentrations were included in the meta-analysis [41,42,43,44,45,46,47,48,49,50,51,52,53,54,55,56,57]. Some studies contributed multiple data points (e.g., data were presented for different age or race/ethnic groups).

### 2.8. Statistical Analyses

The standardized data pairs of the log of reported mean natural food folate intake and blood folate concentrations (*i.e.*, serum/plasma folate and/or RBC folate) and the associated standardized SEMs were used to build the models. In usual regression analysis, the independent variable is assumed to be known without error. In the analysis presented here, however, both the independent variable (*i.e.*, log of reported mean natural food folate intake) and the dependent variable (*i.e.*, log of reported mean blood folate concentration) are estimates and are, therefore, subject to sampling variability. This sampling variability is assumed to be summarized by the standardized SEM associated with each estimate. We used a Bayesian approach to estimate the parameters of the assumed model to reflect the fact that both the independent and dependent variables in our regression model are subject to sampling variability. We illustrate the approach using RBC folate concentrations as the dependent variable in the regression, but an identical method was used to model the serum/plasma folate concentrations outcomes.

Under the Bayesian regression approach, we assumed that the true unknown values of log of the mean RBC folate concentration and natural food folate intake were related by the model:

$$\mu_{ij}^{RBC}=\beta_{0}+\beta_{1}\;\mu_{ij}^{lIntake}+e_{ij}$$

where $\mu_{ij}^{RBC}$ is the unknown true value of the log of the mean RBC folate concentration for the *j*th result in study *i*, $\mu_{ij}^{lIntake}$ is the corresponding unknown true value for the log of the mean natural food folate intake, $e_{ij}$ is an error term reflecting lack of fit of the regression model and $\beta_{0}$ and $\beta_{1}$ are the regression model parameters we wish to estimate and $\beta_{1}$ reflects the level of association between natural food folate intake and RBC folate concentrations, the relationship we are most interested in describing. 

Note that back transforming the model from the log scale results in the nonlinear model:

$$RBC_{ij}=e^{\beta_{0}}*Intake_{ij}^{\beta_{1}}*e^{e_{ij}}$$

where *RBC*_ij_ and *Intake*_ij_ are the untransformed mean blood folate concentration and natural folate intake values, respectively.

To complete the model, we assumed that the observed values for log of the mean natural food folate intake and log of the mean RBC folate concentration (which are the standardized transformations of the data reported in the selected studies) are samples from a Normal distribution with the mean given by $\mu_{ij}^{RBC}$ for RBC folate concentrations and $\mu_{ij}^{lIntake}$ for natural food folate intakes and standard deviations corresponding to the observed transformed SEMs.

### 2.9. Statistical Modeling

Estimates of the regression parameters were derived using Markov Chain Monte Carlo (MCMC) methods using Open BUGS 3.2.2 software [58]. Three sampling chains, with widely dispersed initial values, were run for each model to enable assessment of convergence. The chains were run for 200,000 iterations with the first 100,000 samples discarded as burn-in and every subsequent 10th sample retained to reduce autocorrelation. As a result, the Bayesian estimates, called posterior estimates, for the model parameters were based on 30,000 samples, 10,000 from each of three chains. This collection of estimates, referred to as the posterior distribution, reflects the uncertainty concerning the true value of the model parameters (*i.e.*, natural food folate intake and blood folate concentrations). Posterior distributions for the model parameters, and other related estimates, are summarized using the median of the 30,000 samples and the 95% equal-tailed credible intervals (CrI) which are defined using the 2.5th and 97.5th percentile of the posterior sample.

In addition to the estimated parameters of the regression model, we were also interested in estimating the RBC folate concentrations at specified levels of natural food folate intake. To do this, we used samples from the posterior predictive distribution of RBC folate concentrations [59]. These values can be thought of as a collection of possible values for RBC folate concentrations at the specified levels of natural food folate intake values under the assumed model.

We did not conduct analyses with data from studies that measured RBC folate concentrations with a PBA due to limited data and previously identified assay limitations [60,61,62]. The meta-analysis for serum/plasma folate concentrations was stratified by assay type (MA or PBA) because research has demonstrated PBA limitations in measuring blood folate concentrations, specifically that folate species are differentially recovered according to an individual’s methylenetetrahydrofolate reductase (*MTHFR*) C677T genotype [60,61].

We conducted sensitivity analyses to investigate the impact of including dietary intake assessment bias, random study-level effects, previous exposure to folic acid fortified foods in settings with a mandatory folic acid fortification policy, and outlier studies on the association between natural food folate intake and blood folate concentrations (as described above).

To assess the potential for bias from the use of varied dietary intake assessment tools (*i.e.*, 24 h recalls, food frequency questionnaires, *etc.*), we used an additional set of models in which bias was incorporated into the Bayesian model for the observed natural food folate intake values. For studies reporting the use of 24 h recalls or weighted food records, values were assumed to be underreporting true intake by 20% [63]. Studies that used a food frequency questionnaire were assumed, a priori, to have anywhere between a 50% underestimation and 50% overestimation of true intake [63]. Alternative models were also assessed to evaluate the potential for both unexplained inter-study heterogeneity and increased correlation among multiple data points reported in the same study by incorporating study-level random effects into the assumed model for the true values of natural food folate intake.

Lastly, although we attempted to exclude studies with participants who had been exposed to folic acid (*i.e.*, folic acid fortification or folic acid containing supplements), there is the potential that some participants could have been exposed to folic acid prior to the study and that any depletion/restriction time periods of ≥120 days were still not sufficient to allow blood folate concentrations to acclimate to a natural food folate only diet. Therefore, we conducted sensitivity analyses in which intake data were stratified by the presence of a mandatory fortification policy at the time of data collection as defined by the Food Fortification Initiative [64]. We did not attempt to stratify or exclude studies that allowed voluntary folic acid fortification of specific food products (e.g., the United Kingdom); as there were no reliable sources of the status of voluntary fortification for all countries.

Technical details on the modeling approach, including assumed prior distributions for model parameters, approaches for convergence assessment, and Directed Acyclic Graphs (DAGs) for all models are provided in the Supplementary S2.

## 3. Results

### 3.1. Study Characteristics

The initial search retrieved a total of 11,237 records. After the removal of duplicates, 9224 titles and abstracts were reviewed. A PRISMA record management flow chart is presented (Figure 1).

A total of 36 unique studies were eligible for the systematic review. Because natural food folate intake and blood folate data were not collected for every participant in every study, we had different sample sizes for the number of subjects providing data on intake and blood. Thirty-one Tier 1 studies provided natural food folate intake data and blood folate data on 13,659 and 9144 healthy nonpregnant, nonlactating women aged 12–49 years, respectively, and were eligible for the meta-analysis. Five Tier 2 studies provided natural food folate intake data and blood folate data on 1828 and 2657 participants, respectively (Tables S2 and S3). Four Tier 1 studies (all controlled trials) were conducted after the implementation of mandatory folic acid fortification in the USA, and 27 studies were conducted in countries without, or prior to implementation of, a mandatory policy on folic acid fortification of staple foods [64]. All participants from the included studies reported nonconsumption of folic acid containing supplements or fortified foods during the study period.

Studies from around the world were represented, including Austria, Belgium, Denmark, Finland, France, the Gambia, Germany, Greece, Hungary, India, Italy, Japan, Lebanon, Malaysia, the Netherlands, Nigeria, the Republic of Korea, Spain, Sweden, Thailand, the United Kingdom, and the USA. 

Among Tier 1 studies, there were five controlled trials (intake of natural food folate ranged from 115 to 800 μg DFE/day; duration between 12–15 weeks), two cohort studies [37,41], and 24 cross-sectional studies (intake of natural food folate ranged from 49 to 383 μg DFE/day). There were two controlled trials [65,66] and three cross-sectional studies in Tier 2 [67,68,69].

Of the three main blood folate analytic methods [MA, PBA, liquid chromatography-tandem mass spectrometry (LC-MS/MS)], 11 Tier 1 studies reported using the MA, 20 reported using a PBA (e.g., chemiluminescent immunoassay, radioimmunoassay, *etc.*), and none reported using LC-MS/MS.

Dietary intake assessment methods varied across Tier 1 studies. Twelve studies reported using 24 h recalls (ranging from one day to four days). Four studies reported using weighed food records (ranging from three days to seven days); four studies reported using food records (ranging from three days to seven days); four studies reported using a FFQ; four studies reported doing a feeding study. Two studies reported doing a combination of methods (FFQ and a recall or 24 h recall for two days) [44,48]. One study reported using a brief, self-administered diet history questionnaire [55].

### 3.2. Risk of Bias

Among 14 Tier 1 studies that measured RBC folate concentrations, seven studies had a high risk of bias, seven had a moderate risk of bias and none had a low risk of bias (Tables S4 and S5). For Tier 1 studies that measured serum/plasma folate concentrations, eight were classified as a high risk of bias, 17 with a moderate risk of bias, and three studies were classified with a low risk of bias [30,32,34]. One Tier 2 study had a moderate risk of bias for RBC folate concentrations [67]; a moderate risk of bias for serum/plasma folate concentrations and a high risk of bias for RBC folate concentrations [66]; the remaining Tier 2 studies had a high risk of bias for both outcomes [65,68,69]. Stratification by risk of bias was not done due to limited data eligible for meta-analysis.

### 3.3. RBC Folate Concentrations

**Figure 2:**
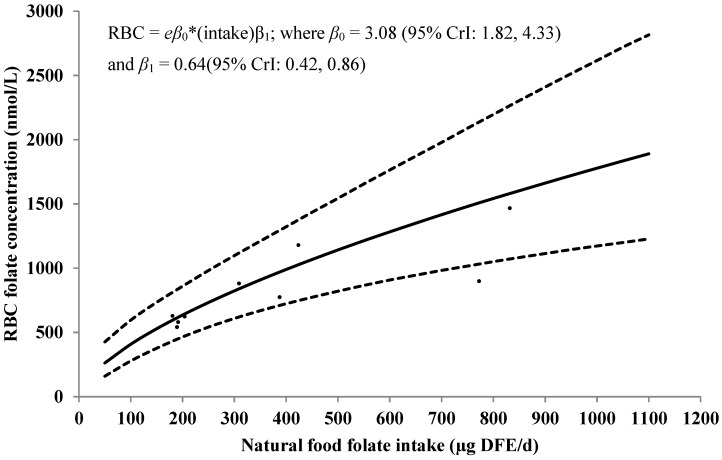
Observed natural food folate intake [μg/day of DFE] *versus* RBC folate concentrations (nmol/L) among women of childbearing age from studies using a MA, no random study effect for intake, and posterior predicted distribution for future values of RBC folate concentration by natural food folate intake. Solid line represents the median value of the posterior predictive distribution; dotted lines represent the 95% CrI. The dots represent the standardized data points from the included studies. Included studies: [31,34,37,38,39,40] CrI: Credible interval; DFE: Dietary folate equivalent; MA: Microbiologic assay; RBC: Red blood cell.

For studies using MA, the estimated association between natural food folate intake and RBC folate concentrations among women aged 12–49 years old is depicted in Figure 2. Under the assumed model, we estimate that a 10% increase in natural food folate intake can increase RBC folate concentrations by approximately 6% (95% CrI: 4%, 9%). Posterior predicted values using the model were consistent with the data presented in the included studies.

Table 1 shows natural food folate intake levels with the corresponding median posterior RBC folate concentration value under the assumed model. For example, using the model, a population in which the mean natural food folate intake was 450 μg DFE/day is estimated to have a mean RBC folate concentration of approximately 1070 nmol/L (95% CrI: 770 , 1440 nmol/L).

### 3.4. Serum/Plasma Folate Concentrations

**Figure 3:**
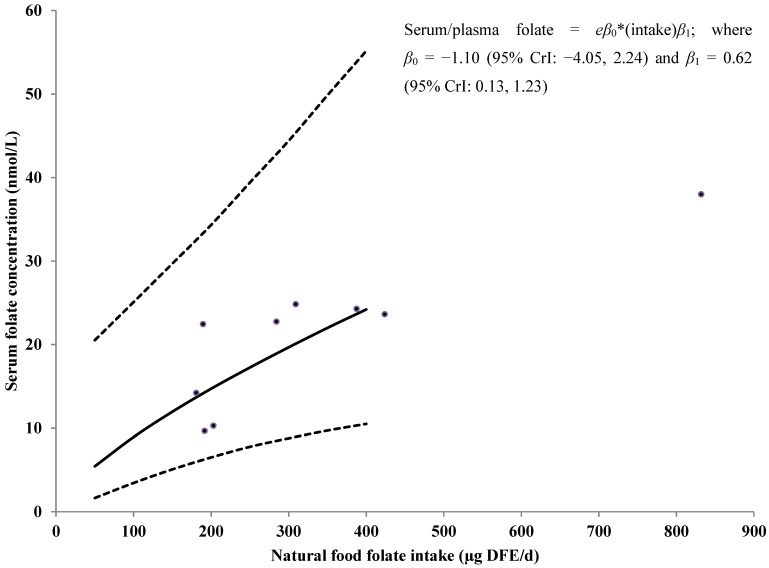
Observed natural food folate intake (μg/day of DFE) *versus* serum/plasma folate concentrations (nmol/L) among women of childbearing age from studies using a MA, no random study effect for intake, and posterior predicted distribution for future values of serum/plasma folate concentration by natural food folate intake. Solid line represents the median value of the posterior predictive distribution; dotted red lines represent the 95% CrI. The dots represent the standardized data points from the included studies. Included studies: [31,34,37,38,39,40]. CrI: Credible interval; DFE: Dietary folate equivalent; MA: Microbiologic assay.

**Figure 4:**
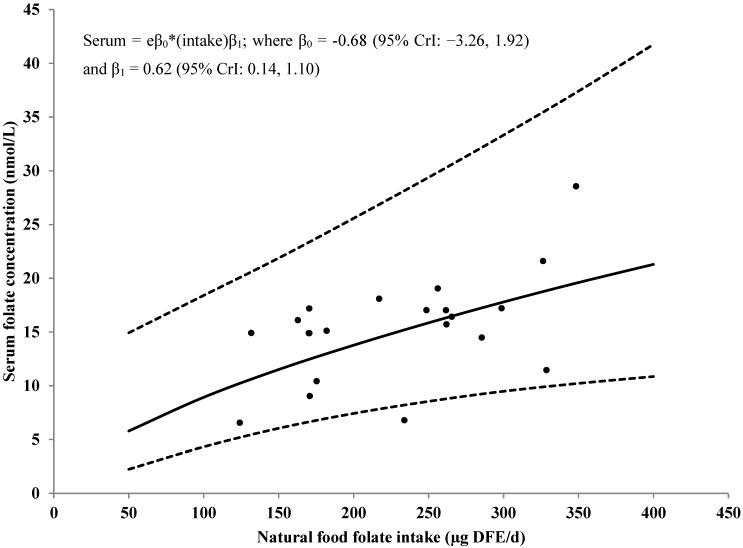
Observed natural food folate intake [μg/day of DFE] *versus* serum/plasma folate concentrations (nmol/L) among studies using a PBA, no random study effect for intake, and posterior predicted distribution for future values of serum/plasma folate concentration by natural food folate intake. Solid line represents the median value; dotted lines represent the 95% CrI. The dots represent the standardized data points from the included studies. Included studies: [41,42,43,44,45,46,47,48,49,50,51,52,53,54,55,56,57]. CrI: Credible interval; DFE: Dietary folate equivalent; PBA: Protein binding assay.

Figure 3 shows the association between natural food folate intake and serum/plasma folate concentrations among women aged 12–49 years old using data from studies that assessed serum/plasma folate concentrations with MA. These results indicate that for every 10% increase in natural food folate intake, serum/plasma folate concentrations could increase by approximately 7% (95% CrI: 1%, 12%). Similar results are seen for studies assessing serum/plasma folate concentrations with PBA (Figure 4).

### 3.5. Sensitivity Analyses

For both the RBC and serum/plasma models, incorporating study-level random effects, and separately, natural food folate intake assessment bias, had no meaningful effect on the predicted association between natural food folate intake and the resulting blood folate concentrations.

Sensitivity analyses assessing the impact of previous exposure to folic acid via fortification indicated that among countries without a mandatory folic acid fortification policy, the estimated slope was slightly higher for both RBC and serum/plasma folate concentrations. This was most likely due to the removal of a study in a fortified setting with high folate intake (800 μg DFE/day) [31]. However, estimates of the slope were fairly consistent across intakes below ~400 μg DFE/day, regardless of the inclusion/exclusion of Hung *et al.* [31].

Lastly, models including/excluding data from two potential outlier studies [35,36] influenced the results dramatically and lowered the estimated slope of the line for both serum/plasma folate and RBC folate results (Supplementary S2: Figures S3 and S4). Although inclusion of these studies did affect model slopes, these differences did not change the overall interpretation of our results.

**Table 1:** Natural food folate intake [μg/day of DFE] and associated RBC folate concentrations (nmol/L) based on Bayesian modeling of association between natural food folate intake and RBC folate concentrations. The shaded values (natural food folate intakes between 450 μg DFE/day and 650 μg DFE/day) refer to the range of intakes and RBC folate concentrations associated with the lowest population risk for a neural tube defect-affected pregnancy according to Crider *et al.* 2014 of 6 neural tube defects per 10,000 live births [6]. Intakes and concentrations above these values may not confer additional neural tube defect prevention benefit.

Natural Food	Median	95% Credible Interval
Folate Intake (μg DFE/day)	RBC Folate (nmol/L)	
50	260	(160, 420)
100	410	(280, 590)
150	530	(380, 730)
200	640	(460, 860)
250	730	(540, 980)
300	820	(610, 1100)
350	910	(670, 1210)
400	990	(720, 1330)
450	1070	(770, 1440)
500	1140	(820, 1550)
550	1220	(880, 1670)
600	1290	(910, 1760)
650	1350	(950, 1870)
700	1420	(980, 1970)
750	1480	(1030, 2080)
800	1550	(1060, 2180)
850	1600	(1080, 2300)
900	1670	(1120, 2390)
950	1730	(1150, 2520)
1000	1780	(1190, 2610)
1050	1840	(1210, 2700)
1100	1900	(1230, 2810)

## 4. Discussion

As far as we are aware, this is the first systematic review and meta-analysis to present estimates of the association between natural food folate alone and blood folate concentrations in a variety of global settings. Overall, we found that natural food folate intake has an impact on both RBC folate and serum/plasma folate concentrations, although the precision of the estimates differ. Using the derived model for data reported using MA, we estimate that a 10% increase in natural food folate intake could lead to an increase in RBC folate concentration of 6% (95% CrI: 4%, 9%). Similarly, for every 10% increase in natural food folate intake, our models show that serum/plasma folate concentrations could increase by 6%–7% regardless of what assay method was used, PBA or MA.

Our results are higher than studies that have assessed the association between total folate intake from mixed dietary sources (natural food folate and folic acid). A meta-analysis by Berti *et al.* using data from mostly nonpregnant, nonlactating women of childbearing age, reported that a doubling in total folate intake, including folic acid, resulted in an increase in RBC folate concentrations by 23% and in serum/plasma folate concentrations by 47% [70]. A meta-analysis by Duffy *et al.* of randomized controlled trials in healthy adults found that blood folate concentrations increased in response to folic acid in a dose-response manner up to 400 μg/day [71]. Among studies with folic acid intakes in the range of 50 μg/day to 400 μg/day, Duffy *et al.* reported that a doubling of folic acid intake resulted in an increase in RBC folate concentrations by 31% (irrespective of assay type) and an increase in serum/plasma folate concentrations by 63% (71% for MA studies; 61% for non-MA studies) [71]. Comparatively, our findings are based on natural food folate intake alone and show higher percentage increases (e.g., a doubling of natural food folate intake would result in a 60% to 70% increase in RBC folate or serum/plasma folate concentrations). This highlights the importance of natural food folate intake in populations in which folic acid is not readily available or consumed (either via fortification of staple foods or as folic acid containing supplements).

In the USA, there are two key recommendations related to folate intake for women of childbearing age. First, the Institute of Medicine (IOM) established the Recommended Dietary Allowance (RDA) for folate at 400 μg DFE/day [2]. This value represents the average daily amount of folate needed to meet the nutrient requirements for 97% to 98% of a healthy population in specific age/gender groups [2]. Second, the US Public Health Service, US Preventive Services Task Force, and the IOM recommend that all women capable of becoming pregnant consume 400 μg/day of folic acid to reduce the risk of having a pregnancy affected by a neural tube defect [2,10,72]. Although these recommendations are critical and remain important public health messages, there has been scant information regarding the dose-response of natural food folate intake to blood folate concentrations, and functionally, to the effectiveness of neural tube defect prevention. Crider *et al.* have modeled an “optimal” RBC folate concentration to prevent neural tube defect-affected births of ≥ about 1050 nmol/L (1180 nmol/L, 95% CrI: 1050 nmol/L, 1340 nmol/L; and have associated it with a neural tube defect risk of approximately 6 per 10,000 live births) [6]. Our meta-analysis results suggest that at the population level, women could achieve the lower bound of this RBC folate concentration range, the “optimal” RBC folate concentration for the prevention of neural tube defects, through a natural food folate intake of at least 450 μg DFE/day. Crider’s model indicates that folic acid intakes at higher levels could further reduce risk; however, there appears to be diminishing returns on neural tube defect risk reduction at RBC folate concentrations above 1300 nmol/L–1500 nmol/L [6].

The Food Fortification Initiative currently estimates that 77 countries report mandatory folic acid fortification legislation for at least one industrially milled cereal grain [64]. This leaves the majority of other countries with limited or no access to folic acid through fortified foods. These populations would need to rely on natural food folate intake or high compliance with periconceptional folic acid supplementation recommendations to reach recommended intake levels for general nutrient requirements (*i.e.*, RDAs) or for neural tube defect prevention [2,10,72]. Though the IOM recommends 400 μg DFE/day as the RDA [2], prior to folic acid fortification in the USA, most of the adult population reported consuming 200–300 μg/day of folate, which would have been predominantly in the form of natural food folate [73]. As seen in our review, outside of a controlled feeding study, most populations did not meet the IOM’s RDA recommendations from natural food folate intake alone. Consuming at least 450 μg DFE/day through food sources alone could be hard to achieve, as intensive education efforts to change dietary habits can be difficult to sustain, and such foods may be seasonal or cost-prohibitive for some populations [74,75,76]. Additionally, about half of all US pregnancies are unplanned [77] and less than a third of US women of childbearing age report consuming a supplement containing folic acid [78]. Therefore, folic acid fortification of staple foods remains an important and critical contributor to meeting total folate needs in the population and reducing the risk of neural tube defect-affected pregnancies. Understanding how natural food folate intake contributes to blood folate concentrations and extrapolating to the model established by Crider *et al.* [6] could potentially inform the identification of neural tube defect risk among populations with limited or no access to folic acid.

This systematic review and meta-analysis has several strengths. First, we stratified our analyses by assay type (MA or PBA) and limited RBC folate concentration models to studies utilizing MA only. This decision was based on previous research indicating that whole blood folate concentrations measured using the BioRad Quantaphase II radioimmunoassay (BRQ II RIA, a type of PBA) differentially recover folate species according to an individual’s methylenetetrahydrofolate reductase (*MTHFR*) C677T genotype [61]. A systematic review found that such results for RBC folate concentrations are not limited to the BRQ II RIA, but extend to other types of PBA [14]. While serum/plasma folate concentrations have not been shown to be as biased by genotype as are RBC folate concentrations [61,79], the within and between person variability should be considered when interpreting the impact of natural food folate intake on serum/plasma folate concentrations, and stratification by assay type is still an important consideration.

Second, data in this review represent reported natural food folate intake and blood folate concentrations from populations around the world. Study authors were contacted for additional information, and based on this information and to the best of the authors’ knowledge, no participants reported any consumption of folic acid. Third, the flexibility of the Bayesian modeling approach allowed us to account for the fact that both the dependent variable (blood folate concentration) and the independent variable (natural food folate intake) in the assumed regression model were subject to varying levels of sampling variability. Fourth, efforts were made to standardize measures of central tendency and MA methods to increase comparability across studies. Fifth, given the specified levels of natural food folate intake, the possible values for folate concentration produced using the model were consistent with the collection of observed values in the selected studies. Lastly, we conducted a series of sensitivity analyses to assess the impact of different factors on the model-based predictions, including study-level random effects, the potential for bias in reported measures of natural food folate intake, previous consumption of folic acid fortified foods in countries with a mandatory fortification policy, and the exclusion of potential outlier studies. Overall, these sensitivity analyses did not change the interpretation of our findings.

This study was also subject to several limitations. First, there was the potential that blood folate concentrations still reflected consumption of folic acid among participants in feeding studies conducted in countries with mandatory folic acid fortification policies. These studies often had depletion phases that were not long enough to bring RBC folate concentrations down to pre-fortification concentrations. However, depletion phase data that were less than 120 days were excluded from the meta-analysis. Second, we did not delineate voluntary fortification status because access to voluntarily fortified foods is difficult to assess and often unregulated by governments. Therefore, blood folate concentration data from some studies could have reflected some folic acid consumption if voluntarily fortified foods were consumed by the study population. However, to the best of the authors’ knowledge, study participants did not report consumption of fortified foods. Third, dietary intake methods have varying levels of measurement error [3]; thus, as research has shown, the assessment of natural food folate intake will also be subject to measurement error [80]. Park *et al.* reported multiple issues when assessing folate intake including error associated with the measurement tool, the food composition database used, and the seasonality of data collection [80]. These are serious limitations to our analysis; however, these issues were directly related to the study design of included studies and could not be altered for purposes of this analysis. We were unable to account for both the seasonality of data collection and the food composition database used in each study; however, we attempted to model the measurement error for different dietary intake assessment methods by incorporating varying levels of bias associated with each measurement tool into the Bayesian model (e.g., under-reporting for 24 h recall and under- and over-reporting for food frequency questionnaires). This approach was chosen because stratification by dietary assessment intake method reduced our sample size, and we could not be sure that each assessment method had been implemented in a similar fashion across studies. Additionally, assessment methods and the implementation of these methods could have improved over time. The Bayesian model allowed us to increase the error around our estimates of natural food folate intake. Even with the increased error around these estimates, we still found an association between natural food folate intake and blood folate concentrations. Fourth, study time periods were not always long enough to ensure that RBC folate status could change based on natural food folate intake alone. Fifth, studies in countries without a mandatory folic acid fortification policy tended to use the PBA method and were cross-sectional, so we were unable to conduct a comparison of studies in fortifying countries by assay and study design. Sixth, the *MTHFR* C677T genotype was not accounted for because not all studies genotyped their participants, so we were unable to conclude whether the association between natural folate intake and blood folate concentrations differed by genotype. Seventh, studies that used PBA were grouped together, although there are known differences among different types of PBA methods [24,25]. Eighth, our results demonstrate wide credibility intervals and are generalizable only across the range of intakes and race/ethnic groups represented by the data from the studies included in the analyses. Lastly, because of the limited data available for the meta-analyses, we were not able to stratify on certain variables (e.g., by dietary assessment method) and a single study has the potential to change the observed associations. However, to address the latter, we conducted sensitivity analyses with potential influential outlier studies and the interpretation of our results did not change.

## 5. Conclusions

A 10% increase in natural food folate intake is associated with a 6% to 7% increase of both serum/plasma and RBC folate concentrations. This information is critical for countries with limited or no access to folic acid through fortification or folic acid supplementation. Our results, when interpreted alongside models assessing RBC folate concentrations and the risk of neural tube defects, estimate that women could possibly achieve the lower bound of a suggested “optimal” RBC folate concentration with a consumption of at least 450 μg DFE/day. However, to reach this value requires careful planning and long-term adoption of effective dietary interventions. Prevention efforts that do not rely on behavior change, such as fortification of staple cereal grain products with folic acid, could allow women to more easily consume adequate total folate in their diet, resulting in increased blood folate concentrations and reduced risk for a neural tube defect-affected pregnancy.

## Supplementary Files

Supplementary File 1

## References

[1] National Institutes of Health: Office of Dietary Supplements Folate. http://ods.od.nih.gov/factsheets/Folate-HealthProfessional/.

[2] Institute of Medicine (1998). Dietary Reference Intakes for Thiamin, Riboflavin, Niacin, Vitamin B6, Folate, Vitamin B12, Pantothenic Acid, Biotin, and Choline.

[3] Gibson R.S. (2005). Principles of Nutritional Assessment.

[4] World Health Organization (2013). Serum and Red Blood Cell Folate Concentrations for Assessing Folate Status in Populations.

[5] Daly L.E., Kirke P.N., Molloy A., Weir D.G., Scott J.M. (1995). Folate levels and neural tube defects: Implications for prevention. JAMA.

[6] Crider K.S., Devine O., Hao L., Dowling N.F., Li S., Molloy A.M., Li Z., Zhu J.-H., Berry R.J. (2014). Population red blood cell folate concentrations for prevention of neural tube defects: Bayesian model. BMJ.

[7] Pfeiffer C., Hughes J.P., Lacher D.A., Bailey R.L., Berry R.J., Zhang M., Yetley E.A., Rader J.I., Sempos C.T., Johnson C.L. (2012). Estimation of trends in serum and RBC folate in the U.S. population from pre- to postfortification using assay-adjusted data from the NHANES 1988–2010. J. Nutr..

[8] Crider K.S., Zhu J.-H., Hao L., Yang Q.-H., Yang T.P., Gindler J., Maneval D.R., Quinlivan E.P., Li Z., Bailey L.B. (2011). *MTHFR* 677→T genotype is associated with folate and homocysteine concentrations in a large, population-based, double-blind trial of folic acid supplementation. Am. J. Clin. Nutr..

[9] Berry R.J., Li Z., Erickson J.D., Li S., Moore C.A., Wang H., Mulinare J., Zhao P., Wong L.Y., Gindler J. (1999). Prevention of neural-tube defects with folic acid in China. China-U.S. collaborative project for neural tube defect prevention. N. Engl. J. Med..

[10] Centers for Disease Control and Prevention (1992). Recommendations for the use of folic acid to reduce the number of cases of spina bifida and other neural tube defects. MMWR Recomm. Rep..

[11] MRC Vitamin Study Research Group (1991). Prevention of neural tube defects: Results of the Medical Research Council Vitamin Study. Lancet.

[12] Centers for Disease Control and Prevention (2010). CDC grand rounds: Additional opportunities to prevent neural tube defects with folic acid fortification. MMWR Morb. Mortal. Wkly. Rep..

[13] Williams L.J., Rasmussen S.A., Flores A., Kirby R.S., Edmonds L.D. (2005). Decline in the prevalence of spina bifida and anencephaly by race/ethnicity: 1995–2002. Pediatrics.

[14] Tsang B.L., Devine O.J., Cordero A.M., Marchetta C.M., Mulinare J., Mersereau P., Guo J., Qi Y.P., Berry R.J., Rosentha J. (2015). Assessing the association between the methylenetetrahydrofolate reductase (MTHFR) 677C>T polymorphism and blood folate concentrations: A systematic review and meta-analysis of trials and observational studies. Am. J. Clin. Nutr..

[15] Higgins J.P.T., Green S. (2011). Cochrane Handbook for Systematic Reviews of Interventions.

[16] Moher D., Liberati A., Tetzlaff J., Altman D.G., Group P. (2009). Preferred reporting items for systematic reviews and meta-analyses: The PRISMA statement. J. Clin. Epidemiol..

[17] Viswanathan M., Berkman N.D. (2012). Development of the RTI item bank on risk of bias and precision of observational studies. J. Clin. Epidemiol..

[18] Souverein O.W., Dullemeijer C., van’t Veer P., van der Voet H. (2012). Transformations of summary statistics as input in meta-analysis for linear dose-response models on a logarithmic scale: A methodology developed within EURRECA. BMC Med. Res. Methodol..

[19] Pfeiffer C.M., Zhang M., Lacher D.A., Molloy A.M., Tamura T., Yetley E.A., Picciano M.F., Johnson C.L. (2011). Comparison of serum and red blood cell folate microbiologic assays for national population surveys. J. Nutr..

[20] Tamura T., Picciano M.F., Stokstad E.L.R. (1990). Microbiological assay of folates. Folic Acid Metabolism in Health and Disease.

[21] O’Broin S., Kelleher B. (1992). Microbiological assay on microtitre plates of folate in serum and red cells. J. Clin. Pathol..

[22] Molloy A.M., Scott J.M. (1997). Microbiological assay for serum, plasma and red cell folate using cryopreserved, microtiter plate method. Methods Enzymol..

[23] Rothman K., Greenland S., Lash T. (2012). Modern Epidemiology.

[24] Gunter E.W., Bowman B.A., Caudill S.P., Twite D.B., Adams M.J., Sampson E.J. (1996). Results of an international round robin for serum and whole-blood folate. Clin. Chem..

[25] Owen W.E., Roberts W.L. (2003). Comparison of five automated serum and whole blood folate assays. Am. J. Clin. Pathol..

[26] Satija A., Yu E., Willett W.C., Hu F.B. (2015). Understanding nutritional epidemiology and its role in policy. Adv. Nutr..

[27] Chew S.C., Khor G.L., Loh S.P. (2011). Association between dietary folate intake and blood status of folate and homocysteine in Malaysian adults. J. Nutr. Sci. Vitaminol..

[28] Rasmussen L.B., Ovesen L., Bulow I., Knudsen N., Laurberg P., Perrild H. (2000). Folate intake, lifestyle factors, and homocysteine concentrations in younger and older women. Am. J. Clin. Nutr..

[29] Agodi A., Barchitta M., Quattrocchi A., Marchese A.E., Boffetta P. (2014). Folate deficiency is not associated with increased mitochondrial genomic instability: Results from dietary intake and lymphocytic mtDNA 4977-bp deletion in healthy young women in Italy. Mutagenesis.

[30] Abratte C.M., Wang W., Li R., Moriarty D.J., Caudill M.A. (2008). Folate intake and the MTHFR C677T genotype influence choline status in young Mexican American women. J. Nutr. Biochem..

[31] Hung J., Yang T.L., Urrutia T.F., Li R., Perry C.A., Hata H., Cogger E.A., Moriarty D.J., Caudill M.A. (2006). Additional food folate derived exclusively from natural sources improves folate status in young women with the MTHFR 677 CC or TT genotype. J. Nutr. Biochem..

[32] Perry C.A., Renna S.A., Khitun E., Ortiz M., Moriarty D.J., Caudill M.A. (2004). Ethnicity and race influence the folate status response to controlled folate intakes in young women. J. Nutr..

[33] Shelnutt K.P., Kauwell G.P., Chapman C.M., Gregory J.F., Maneval D.R., Browdy A.A., Theriaque D.W., Bailey L.B. (2003). Folate status response to controlled folate intake is affected by the methylenetetrahydrofolate reductase 677C→T polymorphism in young women. J. Nutr..

[34] Wright A.J., King M.J., Wolfe C.A., Powers H.J., Finglas P.M. (2010). Comparison of (6 S)-5-methyltetrahydrofolic acid v. folic acid as the reference folate in longer-term human dietary intervention studies assessing the relative bioavailability of natural food folates: Comparative changes in folate status following a 16-week placebo-controlled study in healthy adults. Br. J. Nutr..

[35] Kwanbunjan K., Thepouyporn A., Songmuaeng K., Nakosiri W., Cheeramakara C., Chusongsang Y., Laisupasin P., Tunsakul S., Chantaranipapong Y., Pooudong S. (2008). Food behavior and folate status of hill-tribe schoolchildren and women of childbearing age on the northern border of Thailand. Southeast Asian J. Trop. Med. Public Health.

[36] Pathak P., Saxena R., Kapoor S.K., Dwivedi S.N., Singh R., Kapil U. (2004). Status of serum ferritin and folate levels amongst young women in a rural community of Haryana, India. Nepal Med. Coll. J..

[37] Jang H.B., Han Y.H., Piyathilake C.J., Kim H., Hyun T. (2013). Intake and blood concentrations of folate and their association with health-related behaviors in Korean college students. Nutr. Res. Pract..

[38] Han Y.H., Yon M., Hyun T.H. (2005). Folate intake estimated with an updated database and its association to blood folate and homocysteine in Korean college students. Eur. J. Clin. Nutr..

[39] Kim H.A., Lim H.S. (2008). Dietary folate intake, blood folate status, and urinary folate catabolite excretion in Korean women of childbearing age. J. Nutr. Sci. Vitaminol..

[40] Khor G.L., Duraisamy G., Peng Loh S., Green T.J., Skeaff C.M. (2006). Dietary and blood folate status of Malaysian women of childbearing age. Asia Pac. J. Clin. Nutr..

[41] Dominguez-Salas P., Moore S.E., Cole D., da Costa K.A., Cox S.E., Dyer R.A., Fulford A.J., Innis S.M., Waterland R.A., Zeisel S.H. (2013). DNA methylation potential: Dietary intake and blood concentrations of one-carbon metabolites and cofactors in rural African women. Am. J. Clin. Nutr..

[42] Yang Q., Bailey L., Clarke R., Flanders W.D., Liu T., Yesupriya A., Khoury M.J., Friedman J.M. (2012). Prospective study of methylenetetrahydrofolate reductase (MTHFR) variant C677T and risk of all-cause and cardiovascular disease mortality among 6000 US adults. Am. J. Clin. Nutr..

[43] Costa de Carvalho M.J., Guilland J.C., Moreau D., Boggio V., Fuchs F. (1996). Vitamin status of healthy subjects in Burgundy (France). Ann. Nutr. Metab..

[44] Glew R.H., Williams M., Conn C.A., Cadena S.M., Crossey M., Okolo S.N., VanderJagt D.J. (2001). Cardiovascular disease risk factors and diet of Fulani pastoralists of northern Nigeria. Am. J. Clin. Nutr..

[45] Haddad E.H., Berk L.S., Kettering J.D., Hubbard R.W., Peters W.R. (1999). Dietary intake and biochemical, hematologic, and immune status of vegans compared with nonvegetarians. Am. J. Clin. Nutr..

[46] Liu J.J., Prescott J., Giovannucci E., Hankinson S.E., Rosner B., de Vivo I. (2013). One-carbon metabolism factors and leukocyte telomere length. Am. J. Clin. Nutr..

[47] Sofi F., Innocenti G., Dini C., Masi L., Battistini N.C., Brandi M.L., Rotella C.M., Gensini G.F., Abbate R., Surrenti C. (2006). Low adherence of a clinically healthy Italian population to nutritional recommendations for primary prevention of chronic diseases. Nutr. Metab. Cardiovasc. Dis..

[48] Vandevijvere S., Geelen A., Gonzalez-Gross M., van’t Veer P., Dallongeville J., Mouratidou T., Dekkers A., Bornhorst C., Breidenassel C., Crispim S.P. (2013). Evaluation of food and nutrient intake assessment using concentration biomarkers in European adolescents from the Healthy Lifestyle in Europe by Nutrition in Adolescence study. Br. J. Nutr..

[49] Castetbon K., Vernay M., Malon A., Salanave B., Deschamps V., Roudier C., Oleko A., Szego E., Hercberg S. (2009). Dietary intake, physical activity and nutritional status in adults: The French nutrition and health survey (ENNS, 2006–2007). Br. J. Nutr..

[50] Henriquez P., Doreste J., Diaz-Cremades J., Lopez-Blanco F., Alvarez-Leon E., Serra-Majem L. (2004). Folate status of adults living in the Canary Islands (Spain). Int. J. Vitam. Nutr. Res..

[51] Hiraoka M. (2001). Nutritional status of vitamin A, E, C, B1, B2, B6, nicotinic acid, B12, folate, and beta-carotene in young women. J. Nutr. Sci. Vitaminol..

[52] Hiraoka M. (2004). Folate intake, serum folate, serum total homocysteine levels and methylenetetrahydrofolate reductase C677T polymorphism in young Japanese women. J. Nutr. Sci. Vitaminol..

[53] Al Khatib L., Obeid O., Sibai A.M., Batal M., Adra N. (2006). Folate deficiency is associated with nutritional anaemia in Lebanese women of childbearing age. Public Health Nutr..

[54] Planells E., Sanchez C., Montellano M.A., Mataix J., Llopis J. (2003). Vitamins B6 and B12 and folate status in an adult Mediterranean population. Eur. J. Clin. Nutr..

[55] Watanabe H., Ishida S., Konno Y., Matsumoto M., Nomachi S., Masaki K., Okayama H., Nagai Y. (2012). Impact of dietary folate intake on depressive symptoms in young women of reproductive age. J. Midwifery Womens Health.

[56] Kondo A. (2012). Personal communication.

[57] Lee Y., Krawinkel M. (2011). The nutritional status of iron, folate, and vitamin B-12 of Buddhist vegetarians. Asia Pac. J. Clin. Nutr..

[58] Lunn D., Spiegelhalter D., Thomas A., Best N. (2009). The BUGS project: Evolution, critique and future directions (with discussion). Stat. Med..

[59] Gelman A., Carlin J.B., Stern H.S., Dunson D.B., Vehtari A., Rubin D.B. (2013). Bayesian Data Analysis.

[60] Fazili Z., Pfeiffer C.M., Zhang M. (2007). Comparison of serum folate species analyzed by LC-MS/MS with total folate measured by microbiologic assay and Bio-Rad radioassay. Clin. Chem..

[61] Fazili Z., Pfeiffer C.M., Zhang M., Jain R.B., Koontz D. (2008). Influence of 5,10-methylenetetrahydrofolate reductase polymorphism on whole-blood folate concentrations measured by LC-MS/MS, microbiologic assay, and Bio-Rad radioassay. Clin. Chem..

[62] Molloy A.M., Mills J.L., Kirke P.N., Whitehead A.S., Weir D.G., Scott J.M. (1998). Whole-blood folate values in subjects with different methylenetetrahydrofolate reductase genotypes: Differences between the radioassay and microbiological assays. Clin. Chem..

[63] Willett W. (1998). Nutritional Epidemiology.

[64] Food Fortification Initiative Country Profiles. http://www.ffinetwork.org/.

[65] Brouwer I.A., van Dusseldorp M., West C.E., Meyboom S., Thomas C.M., Duran M., van het Hof K.H., Eskes T.K., Hautvast J.G., Steegers-Theunissen R.P. (1999). Dietary folate from vegetables and citrus fruit decreases plasma homocysteine concentrations in humans in a dietary controlled trial. J. Nutr..

[66] Silaste M.L., Rantala M., Alfthan G., Aro A., Kesaniemi A. (2003). Plasma homocysteine concentration is decreased by dietary intervention. Br. J. Nutr..

[67] Mennen L.I., de Courcy G.P., Guilland J.C., Ducros V., Bertrais S., Nicolas J.P., Maurel M., Zarebska M., Favier A., Franchisseur C. (2002). Homocysteine, cardiovascular disease risk factors, and habitual diet in the French Supplementation with Antioxidant Vitamins and Minerals Study. Am. J. Clin. Nutr..

[68] Nagata C., Shimizu H., Takami R., Hayashi M., Takeda N., Yasuda K. (2003). Soy product intake is inversely associated with serum homocysteine level in premenopausal Japanese women. J. Nutr..

[69] Taguchi T., Mori H., Hamada A., Yamori Y., Mori M. (2012). Serum folate, total homocysteine levels and methylenetetrahydrofolate reductase 677C>T polymorphism in young healthy female Japanese. Asia Pac. J. Clin. Nutr..

[70] Berti C., Fekete K., Dullemeijer C., Trovato M., Souverein O.W., Cavelaars A., Dhonukshe-Rutten R., Massari M., Decsi T., van’t Veer P. (2012). Folate intake and markers of folate status in women of reproductive age, pregnant and lactating women: A meta-analysis. J. Nutr. Metab..

[71] Duffy M.E., Hoey L., Hughes C.F., Strain J.J., Rankin A., Souverein O.W., Dullemeijer C., Collings R., Hooper L., McNulty H. (2014). Biomarker responses to folic acid intervention in healthy adults: A meta-analysis of randomized controlled trials. Am. J. Clin. Nutr..

[72] Wolff T., Witkop C.T., Miller T., Syed S.B. (2009). Folic acid supplementation for the prevention of neural tube defects: An update of the evidence for the U.S. Preventive Services Task Force. Ann. Intern. Med..

[73] Bentley T.G.K., Willett W.C., Weinstein M.C., Kuntz K.M. (2006). Population-level changes in folate intake by age, gender, and race-ethnicity after folic acid fortification. Am. J. Public Health.

[74] Monsivais P., Drewnowski A. (2007). The rising cost of low-energy-density foods. J. Am. Diet. Assoc..

[75] Finzer L.E., Ajay V.S., Ali M.K., Shivashankar R., Goenka S., Sharma P., Pillai D.S., Khandelwal S., Tandon N., Reddy K.S. (2013). Fruit and vegetable purchasing patterns and preferences in South Delhi. Ecol. Food Nutr..

[76] Krajcovicová-Kudlácková M., Valachovicová M., Blazícek P. (2013). Seasonal folate serum concentrations at different nutrition. Cent. Eur. J. Public Health.

[77] Finer L.B., Zolna M.R. (2011). Unintended pregnancy in the United States: Incidence and disparities, 2006. Contraception.

[78] Tinker S.C., Cogswell M.E., Hamner H.C., Berry R.J. (2012). Usual folic acid intakes: A modelling exercise assessing changes in the amount of folic acid in foods and supplements, National Health and Nutrition Examination Survey, 2003–2008. Public Health Nutr..

[79] Molloy A.M., Daly S., Mills J.L., Kirke P.N., Whitehead A.S., Ramsbottom D., Conley M.R., Weir D.G., Scott J.M. (1997). Thermolabile variant of 5,10-methylenetetrahydrofolate reductase associated with low red-cell folates: Implications for folate intake recommendations. Lancet.

[80] Park J.Y., Vollset S.E., Melse-Boonstra A., Chajes V., Ueland P.M., Slimani N. (2013). Dietary intake and biological measurement of folate: A qualitative review of validation studies. Mol. Nutr. Food Res..

