# Supplementary material for: Assessing the Association between Natural Food Folate Intake and Blood Folate Concentrations: A Systematic Review and Bayesian Meta-Analysis of Trials and Observational Studies
Source: Nutrients. 2015 Apr 10;7(4):2663–86. doi: 10.3390/nu7042663 (PMC4425166; doi:10.3390/nu7042663)
Supplement: Supplementary File 1 [file nutrients-07-02663-s001.docx]

Supplementary Information

**Supplementary S1**

**1. Research Question**

What is the association between natural food folate intake and blood folate concentrations among women aged 12–49 years?

**2. Objective**

To perform a systematic review and meta-analysis of the association between natural food folate intake and blood folate concentrations among women aged 12–49 years.

**3. Methods**

*Search strategy*

A research librarian from the CDC Public Health Library will be consulted and will perform the literature search using the following search terms (adapted for databases as necessary) for English language literature published between 1992 December 2011 (The initial search was conducted in April 2012. Given a year had elapsed since our initial search, an updated search of the literature using the same strategy was conducted in July 2013 and again in March 2014 for studies published between January 2012 and March 2014).

We will use the following search strategy to search Embase and adapt as required for other
electronic databases:

1. Folic acid.mp or folic acid/
2. Blood folate.mp or exp folic acid blood level/
3. (Serum folate or plasma folate or red blood cell folate).mp
4. (Intake or diet* or supplement*).mp
5. Folic acid intake.mp
6. Neural tube defect.mp or neural tube defect/
7. Spina bifida.mp or exp spina bifida/
8. Anencephaly.mp or exp anencephalus/
9. (methylenetetrahydrofolate or mthfr).mp
10. (Childbear * or women * or female * or girl * or pregnant *).mp
11. 2 or 3
12. 1 and 4
13. 5 or 12
14. 6 or 7 or 8
15. 10 and 11 and 13
16. 10 and 11 and 14
17. 9 and 10 and 11
18. 10 and 13 and 14
19. 9 and 10 and 13
20. 9 and 10 and 14
21. 15 or 16 or 17 or 18 or 19 or 20
22. Limit 21 to (English language and year = “1992 December 2011”)

In addition to Embase, the following databases will be searched: Cochrane Library, CINAHL, POPLINE, PubMed, and Web of Science

Because these search terms also encompass terms for another concurrent systematic review (the relationship between MTHFR genotype and blood folate concentrations), there will be two screening “waves” that will be used to identify appropriate studies. The first wave will be conducted for both reviews in order to compile a complete list of relevant studies for both research questions. The second screening wave will be specific to each research question and will use more detailed criteria as appropriate.

To identify additional resources, we will search the references of all articles in the full text review.

If age/sex, supplement use stratified data or other population information is needed for eligibility in the review, we will contact authors for additional information.

**4. Wave 1**

*4.1. Inclusion Criteria*

All studies from 1992–March 2014 will be considered, with the exception of case studies:

- Randomized trials, quasi-randomized, and non-randomized control trials with individual or cluster level randomization. Observational designs including cohort, case control, and cross sectional studies will be included.
- Women 12–49 years of age

*4.2. Exclusion Criteria*

Studies that do not contain information on the relationship between at least two of the four following outcome measures:

- - Serum/plasma folate or red blood cell (RBC) folate
  - Folate intake (including all sources from food, supplements, or through a fortified product)
  - Neural tube defect prevalence
  - MTHFR C677T allele or genotype

**5. Wave 2**

*5.1. Inclusion Criteria*

- Females between 12–49 years of age, regardless of baseline folate concentrations, pregnancy status, ethnicity, country of residence.
- In mixed sex/age studies, studies will be included if data for females between 12–49 years of age can be extracted separately, or in which more than half of the participants fulfill this requirement.
- If data specific to females between 12–49 years of age cannot be extracted, studies will be included if the study population is greater than 50% female and between 12–49 years of age and considered second tier quality data.

*5.2. Exclusion Criteria*

- Studies including unhealthy populations, defined as sample recruitment based on a health condition.
- Including, but not limited to: Conditions impacting folate absorption, such as intestinal malabsorption conditions, inflammatory bowel disease, alcohol abuse, cancer, chronic congestive heart failure, chronic renal failure, chronic liver failure, or chronic infectious disease.
- Studies in pregnant, lactating and post-menopausal women.
- Studies without blood folate concentrations stratified by folic acid containing supplement use.
- Studies in populations exposed to folic acid fortification.

*5.3. Selection of Studies*

Excel will be used to store all identified studies from the search strategy. Titles and abstracts will be screened in duplicate. Three teams of two review authors (Heather C. Hamner and Robert J. Berry; Claire M. Marchetta and Jorge Rosenthal; Joe Mulinare and Patricia Mersereau) will review eligibility according to the Wave 1 general search criteria. One team of three (Robert J. Berry, Claire M. Marchetta, and Jorge Rosenthal) will review for eligibility according to Wave 2 criteria specific to this review.

One team of three (Robert J. Berry, Claire M. Marchetta, and Jorge Rosenthal) will also review full
text files.

*5.4. Data Extraction*

For eligible studies, Robert J. Berry, Claire M. Marchetta, and Jorge Rosenthal will independently extract 1/3 of data using a piloted abstraction form. Heather C. Hamner will review for accuracy. We will resolve any discrepancies through discussion and document the process.

The following information will be extracted from each study:

1. Methods

- Study design
- Folate assay method

1. Participants

- Location of the study
- Data collection time frame
- Sample size
- Age
- Blood folate concentrations
- Natural food folate intake
- Method of dietary assessment

1. Intervention (if applicable)

- If relevant, study intervention (e.g., folic acid supplementation)
- Folate intake provided/reported
- Duration of the intervention

Claire M. Marchetta will contact authors identified in full text review for missing information as necessary. Eligible studies will be classified as Tier 1 if all data extracted pertains to non-pregnant or lactating women aged 12–49 years of age. Studies will be classified as Tier 2 if data includes men, or any women outside of the 12–49 age range.

*5.5. Risk of Bias*

Two review authors (Yan Ping Qi, Jing Guo) not included in the search process developed adapted tools for the assessment of risk of bias. For control trials, the *Cochrane Handbook for Systematic Reviews of Interventions* was used [1]. For observational studies, an adapted Item Bank on Risk of Bias and Precision of Observational Studies from the Research Triangle Institute (RTI) was used [2]. Yan Ping Qi Jing Guo will independently evaluate risk of bias or study for the domains below.

5.5.1. Domain Risk of Bias

A score of low, moderate, and high risk of bias score will be assigned to each domain for each study outcome (*i.e.*, serum/plasma folate and RBC folate concentrations). Domains will be considered low risk of bias if all questions within a domain are graded as low, moderate risk of bias if one or more questions are graded as moderate or high risk of bias if one or more questions are graded as high. If we are unable to determine a domain’s risk of bias due to insufficient information provided in a study, the domain will be classified as “unclear” in the risk of bias tables.

5.5.2. Overall Risk of Bias

This same convention will be applied across domains to assign an overall summary score of low, moderate, or high risk of bias for each study outcome (*i.e.*, serum/plasma folate and RBC folate concentrations).

5.5.3. Control Trial Domains

- Random sequence generation
- Allocation concealment
- Blinding of participants and personnel
- Blinding of outcome assessment
- Incomplete outcome bias
- Reporting bias
- Other sources of bias (as needed)

5.5.4. Observational Study Domains

- Uniform and valid measures of inclusion/exclusion criteria
- Appropriateness of study sample
- Exposure and outcome assessment methods
- Appropriate follow-up time (Appropriate follow-up time and completeness of outcome data will not be assessed for cross-sectional studies.)
- Completeness of outcome data (Completeness of outcome data will not be assessed for cross-sectional studies.)
- Adjustment for potential confounding

**6. Data Analysis**

*6.1. Meta-Analyses*

- A statistician (Owen J. Devine) will use Open BUGS 3.2.2 software as statistical software.
- A Bayesian Markov Monte Carlo chain procedure will be used to develop posterior distributions.
  - Each study will be treated as a random effect.
  - The percent change in blood folate concentrations for every doubling of natural food folate intake will be calculated.
- Sensitivity analyses performed will depend on the number of studies eligible for inclusion in the meta-analyses. Anticipated sensitivity analyses of the association of natural food folate intake and blood folate concentrations may be stratified by:
  - Study quality (risk of bias, study tiers)
  - Study design (control trials *vs.* observational studies)
  - Country fortification policy status

**Supplementary S2**

**1. Statistical Methods**

*Data Standardization*

The reported summary measures of central tendency and estimates of the associated variability for natural food folate intake [μg/day dietary folate equivalents (DFE)] and blood folate [serum, plasma, and red blood cell (RBC)] concentrations (nmol/L) varied across papers selected for inclusion in the meta-analysis. For example, some papers reported arithmetic means for untransformed natural food folate intake and blood concentration values, while others summarized their findings using geometric means (reflecting an analysis of log transformed data). In addition, estimates for the sampling variability associated with these summary measures also varied. Some, for example, reported standard deviations, while others reported confidence intervals for means on varying scales. To address these inconsistencies, we transformed all reported values for natural food folate intake and blood folate concentration to the natural log scale, with the resulting inputs to the meta-analysis reflecting the study-specific mean log natural food folate intake and mean log blood folate concentration values and the standard errors of those transformed measures [3].

**2. Regression Models**

*2.1. Natural Food Folate Intake and Red Blood Cell Folate Concentrations*

Abstracted values for natural food folate intake and RBC folate concentrations were examined graphically to identify candidate forms for regression models relating these measures. Based on this assessment, a linear model relating the mean of the log transformed natural food folate intake and the mean of the log transformed RBC folate concentration was deemed acceptable (Figure S1).

We used a Bayesian approach to estimate the parameters of the regression model to address the issue that both the dependent and independent variables in the model, log RBC concentration and log natural food folate intake respectively, are subject to sampling variability. Let *lIntake_ij_* be the observed mean log natural food folate intake for the *j*th result reported in study *i*. To reflect the sampling variability associated with this measure, we assume that

| $lIntake_{ij} \sim N(\mu_{ij}^{lIntake} , \sigma_{ij}^{2}(lIntake) )$ |  |
| --- | --- |

where μ*lIntake ij* is the unknown true value for mean log natural food folate intake among women reported in the *j*th result of study *i* and σ2 *ij*(*lIntake*) is the reported squared standard error associated with the estimate *lIntake_ij_*. In other words, we consider the reported mean log natural food folate intake, *lIntake_ij_*, to be an unbiased estimate of the true mean log natural food folate intake, μ*lIntake ij*, with a sample variance corresponding to the reported value of σ2 *ij*(*lIntake*). For the next level of model hierarchy, we assume that the μ*lIntake ij* are exchangeable, given a population level mean and variance for the log natural food folate intake such that

| $\mu_{ij}^{lIntake} \sim N( \mu^{lIntake}, \sigma^{2}(lIntake) )$ |  |
| --- | --- |

where μ*^lIntake^* is the unknown mean log transformed natural food folate intake among all possible studies and the variation of studies about this mean is represented by the unknown parameter σ^2^(*lIntake*). We next assume that the corresponding reported mean log RBC folate concentration, *lRBC_ij_*, is a sample from a normal distribution such that

| $lRBC_{ij} \sim N(\mu_{ij}^{RBC} , \sigma_{ij}^{2}(RBC) )$ |  |
| --- | --- |

where σ2 *ij*(*RBC*) is the reported squared standard error of *lRBC_ij_* and μ*RBC ij* is the unknown true mean log RBC concentration for result *j* in study *i*. We assume that μ*RBC ij* follows the regression model

| $\mu_{ij}^{RBC} = \beta_{0} + \beta_{1} \mu_{ij}^{lIntake} + e_{ij}$ |  |
| --- | --- |

in which β_0_ and β_1_ are unknown parameters and

|  |  |
| --- | --- |

where σ^2^(*REG*) reflects the residual variance of the true unknown value for mean log RBC folate concentration about β_0_ + β_1_ μ*lIntake ij*. Notice that the parameter β_1_ is the estimand of interest, reflecting
the association between the true mean log natural food folate intake, μ*lIntake ij* and the true mean log
RBC concentration.

| 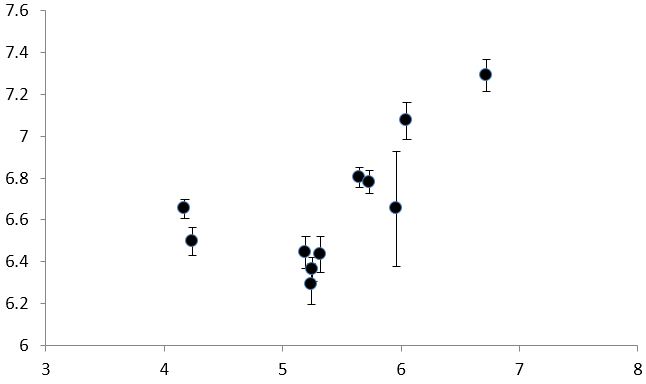 |
| --- |
| (**A**) |

**Figure S1.** *Cont.*

| 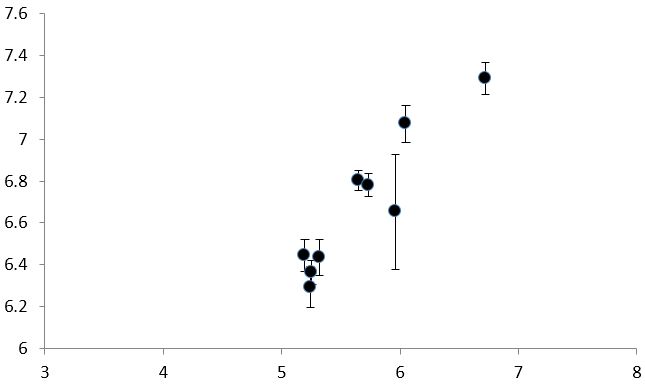 |
| --- |
| (**B**) |

**Figure S1.** Reported mean log red blood cell (RBC) folate concentration using microbiologic assay, with estimated 95% confidence intervals, by mean log natural food folate intake for (**A**) Kwanbunjan *et al*. [4] (2008) included and (**B**) Kwanbunjan *et al.* [4] (2008) excluded.

Completing the specification of the Bayesian model, we assume the following weakly informative prior distributions for the unknown model parameters

| $\mu^{lIntake} \sim N ( 5 , 1000 )$ |  |
| --- | --- |
| $\sigma^{2}(lIntake) \sim U [ 0 , 100 ]$ |  |
|  |  |
| and  |  |
| $\sigma^{2}(REG) \sim U [ 0 , 100 ]$ |  |

where *U*[*a*, *b*] denotes the uniform distribution with lower and upper bounds defined by *a* and *b* respectively. A directed acyclic graph (DAG) summarizing the assumed model relating the reported natural food folate intake and RBC folate concentration is provided in Figure S2.


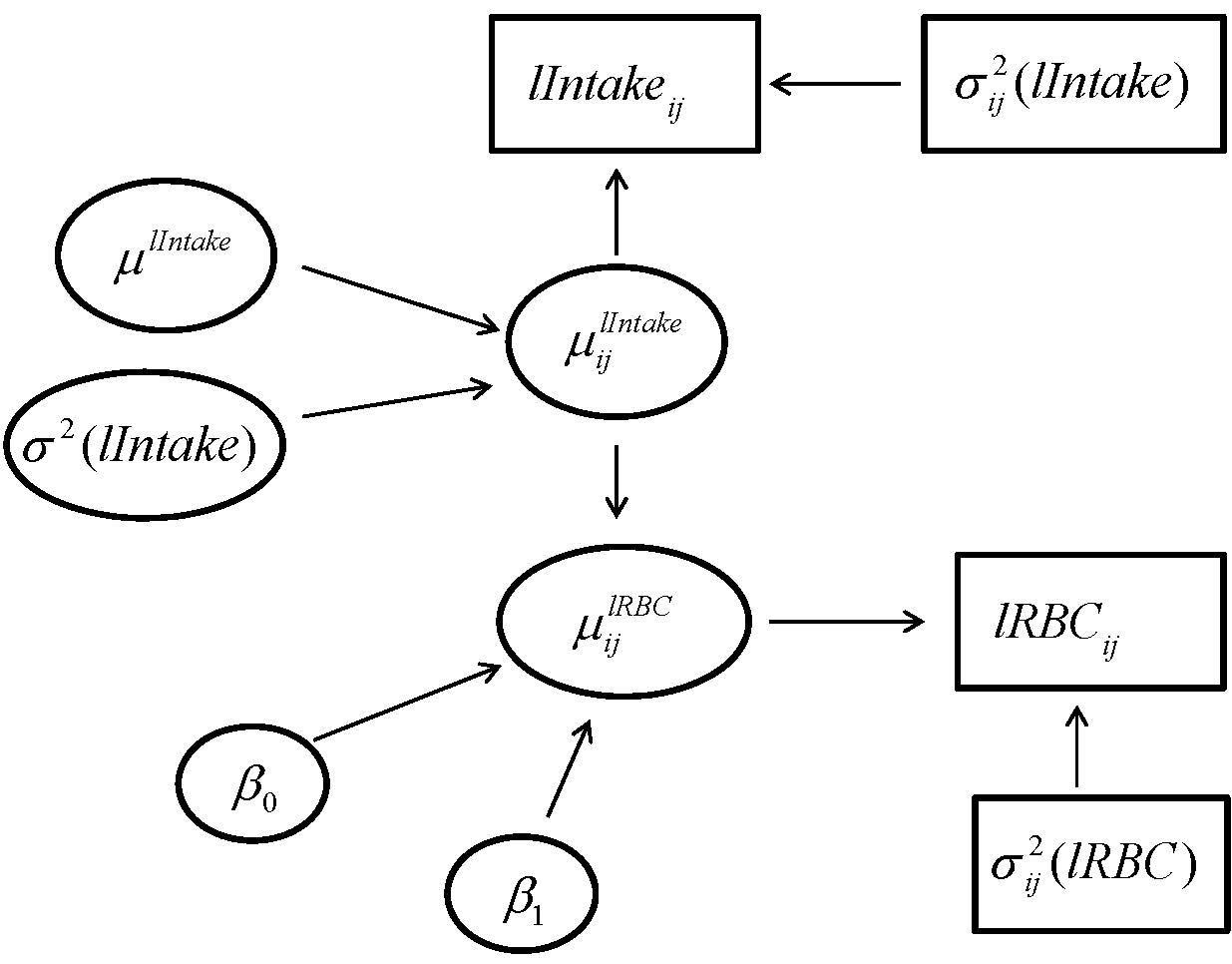


**Figure S2.** Directed acyclic graph (DAG) for analysis model relating observed values of the mean log natural folate *lIntake_ij_* for the *j*th result in study *i* and the corresponding log red blood cell (RBC) folate concentration, *lRBC_ij_*. Note: Values contained in rectangular shapes represent observed quantities while those in circular shapes reflect unknown model parameters.

*2.2. Natural Food Folate Intake and Serum/Plasma Folate Concentrations*

Exploratory analysis for serum/plasma folate concentration, ascertained using both microbiologic and protein binding assays, indicated that an assumption of a linear relationship between the mean of the log reported natural food folate intake and the mean of the log serum/plasma folate concentration appeared reasonable (Figures S3 and S4). As a result, the same form of the model was assumed for the reported serum/plasma folate concentration outcomes as was used for RBC folate concentration.

| 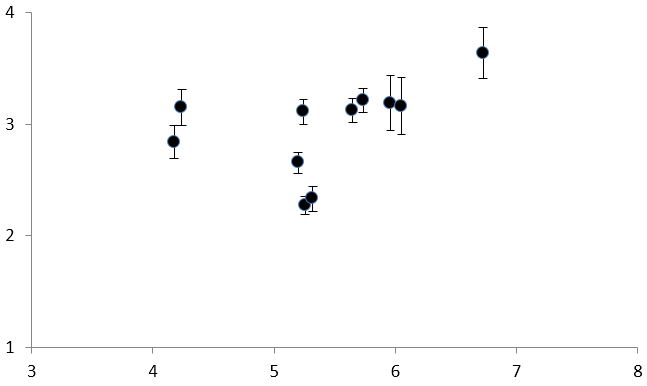 |
| --- |
| (**A**) |
| 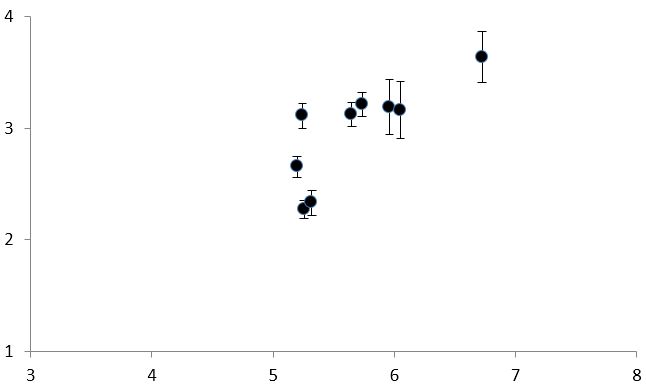 |
| (**B**) |

**Figure S3.** Reported mean log serum/plasma folate concentration using microbiologic assay, with estimated 95% confidence intervals, by mean log natural food folate intake for
(**A**) Kwanbunjan *et al.* [4] (2008) included and (**B**) Kwanbunjan *et al.* [4] (2008) excluded.

| 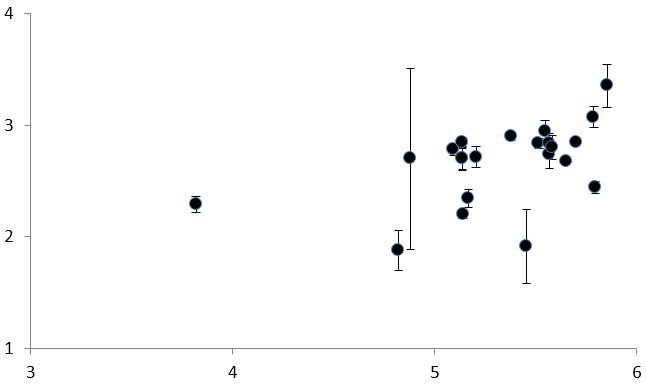 |
| --- |
| (**A**) |
| 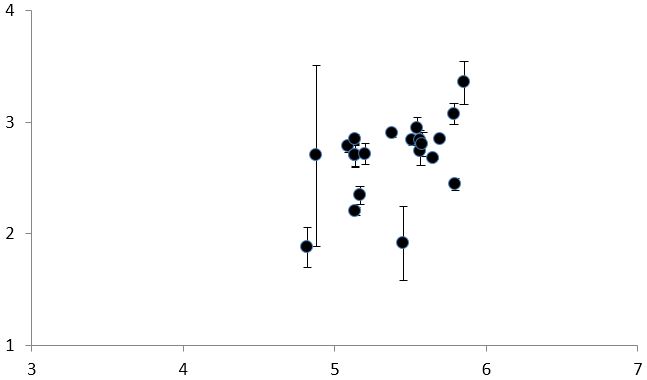 |
| (**B**) |

**Figure S4.** Reported mean log serum/plasma folate concentration using protein binding assay, with estimated 95% confidence intervals, by mean log natural food folate intake for (**A**) Pathak *et al.* [5] (2004) included and (**B**) Pathak *et al.* [5] (2004) excluded.

**3. Exclusion of Outliers**

In the natural food folate intake and blood folate concentration (serum/plasma and RBC folate) analysis for studies using the microbiologic assay, the results reported by Kwanbunjan *et al.* [4] were far from
the collection of other reported intake/blood folate concentration pairs. Due to their distance from the collection of other data, inclusion of serum/plasma and RBC folate concentrations from this study were highly influential. Since subject matter experts also deemed the values of suspect plausibility, we removed these data from the analysis. In addition, in the assessment of the association between natural food folate intake and serum/plasma folate for studies using protein-binding assays, the results of Pathak *et al.* [5] also substantially differed from the cloud of other data and deemed not likely to be plausible. As a result, these results were also omitted from the analysis.

**4. Alternative Models**

As a sensitivity assessment, alternative models were evaluated to determine the impact of assumptions on the model form for (1) the values of the estimated parameters and (2) the resulting estimates of blood folate concentration, given natural food folate intake. The first alternative model incorporated random study-level effects for natural food folate intake, to reflect the possibility of unaccounted for inter-study heterogeneity and for increased correlation among results reported within the same study. Under this alternative model, the observed mean log natural food folate intake is again assumed to follow a normal distribution with expectation defined by the true unobserved study-specific mean log concentration

| $lIntake_{ij} \sim N(\mu_{i}^{lIntake} , \sigma_{ij}^{2}(lIntake) )$ |  |
| --- | --- |

Study-level effects are incorporated in this model by assuming

| $\mu_{i}^{lIntake} = \mu^{lIntake} + \lambda_{i}$ |  |
| --- | --- |

where λ*_i_* is a random effect such that

| $\lambda_{i} \sim N ( 0 , \sigma^{2}(\lambda) )$ |  |
| --- | --- |

In the above model, the standard deviation of the random effects, σ(λ), reflects the magnitude of
inter-study heterogeneity and also reflects a potential increase in the level of correlation among observed intakes in the same study above that in the model without study-level random effects. A uniform prior distribution bounded by 0 and 100 was assumed for σ(λ) A DAG for this model is provided in Figure S5.


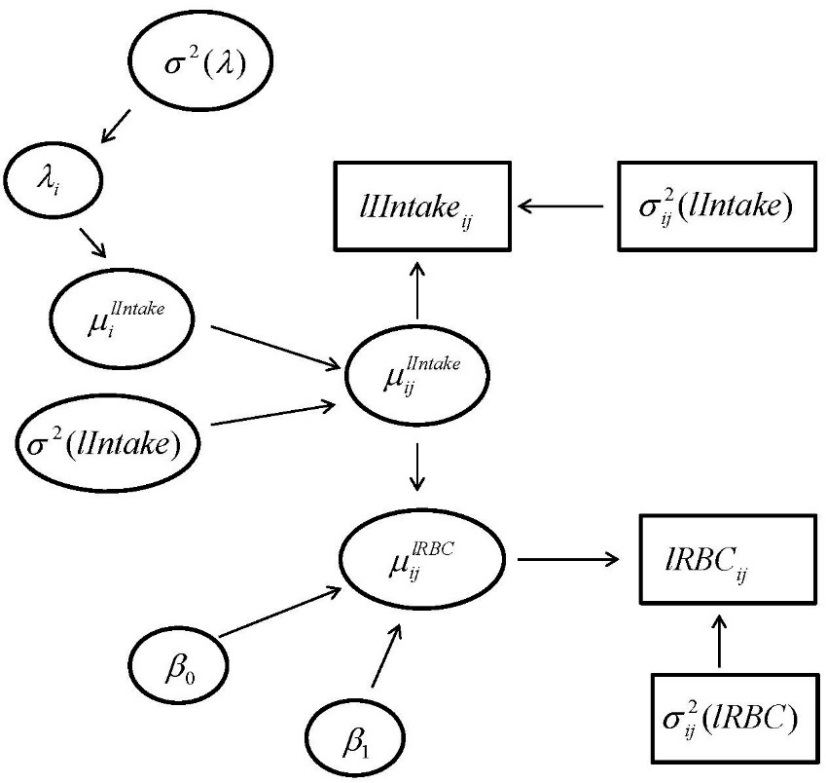


**Figure S5.** Directed acyclic graph (DAG) for analysis model, relating observed values of the mean log natural folate *lIntake_ij_* for the *j*th result in study *i* and the corresponding log red blood cell (RBC) folate concentration, *lRBC_ij_*, assuming study-level random effects. Note: Values contained in rectangular shapes represent observed quantities while those in circular shapes reflect unknown model parameters.

We developed a second alternative model to address the possibility of bias in the reported measures of natural folate intake due to the tool used to estimate these values. For this model, we assumed that reported natural food folate values estimated using either 24-h recall or weighted food records had
a potential underreporting bias of up to 20% less than the true natural food folate intake value [6].
 For studies that used food frequency questionnaires, we assumed a wide range of potential bias from a 50% underestimation to a 50% overestimation of true intake. For studies using dietary assessment tools other than a 24-h recall, weighted food record, or food frequency questionnaires (e.g., controlled feeding trial), the observed log mean natural food folate intake was assumed to follow the original model

| $lIntake_{ij} \sim N(\mu_{ij}^{lIntake} , \sigma_{ij}^{2}(lIntake) )$ |  |
| --- | --- |

Alternatively, observed intake measures reported from studies that used one of these three measurement tools were assumed to follow the model

| $lIntake_{ij} \sim N(\mu_{ij}^{lIntake} + \delta_{i} , \sigma_{ij}^{2}(lIntake) )$ |  |
| --- | --- |

where δ*_i_* represents a study-level bias term altering the expectation of the observed natural food
folate intake. To reflect prior assumptions on the magnitude of this bias, we assumed δ*_i_* to have the prior distribution

| $e^{\delta_{i}} \sim U [ 0.8 , 1.0 ]$ |  |
| --- | --- |

for studies using 24-recal and weighted food records and

| $e^{\delta_{i}} \sim U [ 0.5 , 1.5 ]$ |  |
| --- | --- |

for studies using food frequency questionnaires for intake estimation. A DAG outlining the model under assumed bias in the observed values of mean log folate intake is given in Figure S6.

**5. Model Fitting**

Estimates of the posterior distributions of the model parameters were developed via Markov Chain Monte Carlo (MCMC) simulation using OpenBUGS 3.2.2 software. Three sampling chains were run for each estimation, with widely separated initial values for the model parameters. Chains were run for 200,000 iterations, with the first 100,000 samples discarded to increase the likelihood of convergence to the posterior distribution. To reduce autocorrelation, every 10th sample from the 100,000th through the 200,000th iterations was retained for estimation of the posterior. As a result, estimates for the posterior distribution for model parameters were based on 30,000 samples (10,000 from each of three chains). Convergence was assessed using visual inspection of both history and Gelman-Rubin plots. Posterior estimates of model parameters are summarized using the median value of the 30,000 posterior samples and equal tailed 95% credible intervals defined by the 2.5th and 97.5 percentiles of the collection of samples.


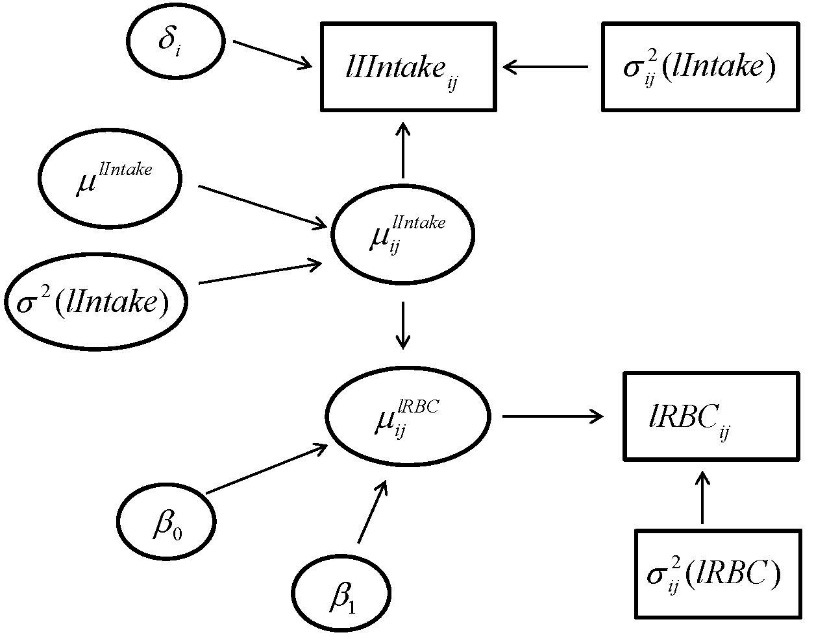


**Figure S6.** Directed acyclic graph (DAG) for analysis model, relating observed values of the mean log natural folate *lIntake_ij_* for the *j*th result in study *i* and the corresponding log red blood cell (RBC) folate concentration, *lRBC_ij_*, assuming bias in observed measures of log mean natural food folate intake. Note: Values contained in rectangular shapes represent observed quantities while those in circular shapes reflect unknown model parameters.

**6. Posterior Predictive Distribution for Folate Concentrations at Specified Levels of Natural Food Folate Intake**

Samples from the posterior predictive distribution of both RBC and serum/plasma folate concentrations at given levels of natural food folate intake were developed based on the posterior samples of model parameters. For example, given a specified natural food folate intake of *I* μg/day DFE, the *i*th sample from the posterior predictive distribution for the associated RBC was generated as *e^lRBCi^* where *lRBC_i_* is generated from the normal distribution

| $lRBC_{i} \sim N( \beta_{0i} + \beta_{1i} \log(I) , \sigma^{2}(REG)_{i})$ |  |
| --- | --- |

and β_0_*_i_*, β_1_*_i_* and σ^2^(*REG*)*_i_* are the *i*th posterior samples, *i* = 1, …, 30,000 of the model parameters.

**Supplementary S3**

**Supplementary Tables**

**Table S1.** Risk of bias assessment tool questions by study design, as adapted from the Item Bank for Assessment of Risk of Bias and Precision for Observational Studies [2].

| **Question followed by criteria for ranking each item as low, medium or high risk of bias** | **Cohort** | **Cross-sectional** |
| --- | --- | --- |
| *Clear inclusion/exclusion criteria:*   1. *Does the article clearly state its own inclusion/exclusion criteria (i.e., does not require the reader to infer)?*   **Low**: Clearly states and defines (as appropriate) excluded groups  **Moderate**: Criteria partially stated or stated but not adequately defined (e.g., states that only healthy subjects are included but does not define healthy)  **High**: Criteria not stated | X | X |
| *Appropriate sample selection:*   1. *Is the sample appropriate?*   **Low**: Population-based or systematic random sample  **High**: No information on how sample was obtained | X | X |
| *Valid assessment of inclusion/exclusion:*   1. *Are the inclusion/exclusion criteria measured using valid and reliable measures?*   **Low**: Physical exam, lab test, or other measure to verify “healthy, pregnancy status, or women of childbearing age ” status; survey of nationally-representative sample  **Moderate**: Self-report (questionnaire or interview); stated as healthy population but undefined  **High**: Inappropriate tool used in assessment of criteria | X | X |

**Table S1.** *Cont.*

| *Valid assessment of exposure:*   1. *Is the level of detail in describing the intervention or exposure adequate?*   **Low**: Provided intake assessment method (*i.e.*, 3d food record, 24 h recall, Food Frequency Questionnaire (FFQ), food composition table, *etc*)  **Moderate**: --  **High**: No information provided   1. *Is intake (the exposure) assessed using valid and reliable measures, implemented consistently across all study participants?*   **Low**: Feeding study  **Moderate**: Food record, 24 h recall, FFQ, or if US food composition tables were used to estimate intake for another country  **High**: Non-validated food intake questionnaire | X | X |
| --- | --- | --- |
| *Valid assessment of outcome:*   1. *Are outcomes (blood folate concentrations) assessed using valid and reliable measures, implemented consistently across all study participants?*   **Low**: Microbiologic assay for serum/plasma and/or red blood cell (RBC) folate.  **Moderate**: Any protein-binding assay for serum/plasma folate. Non Bio-Rad Quantaphase II protein-binding assays for RBC folate.  **High**: Bio-Rad Quantaphase II radioimmunoassay for RBC folate; unknown assay | X | X |
| *Incomplete outcome data:*   1. *Is the length of follow-up the same for all groups?*   **Low**: Yes  **Moderate**:--  **High**: No   1. *Did attrition differ between groups by more than 20%?*   **Low**: No  **Moderate**:--  **High**: Yes   1. *Is the length of follow-up time sufficient to support the evaluation of primary outcomes and harms?*   **Low**: Yes, if >1 day for serum folate and >120 days for RBC folate  **Moderate**:--  **High**: No, if <1 day for serum folate and <120 days for RBC folate | X | N/A |

**Table S1.** *Cont.*

| 1. *Were the important confounding and effect modifying variables taken into account in the design and/or analysis (e.g., through matching, stratification, interaction terms, multivariate analysis, or other statistical adjustment)?*   **Low**: Homogenous sample with additional adjustment for age, race, smoking, caffeine use, alcohol use and body mass index (BMI); heterogeneous sample adjusted for or stratified by race/ethnicity  **Moderate**: Homogenous sample with no adjustment; heterogeneous sample with <10% of any individual minority population  **High**: Heterogeneous sample with no adjustment for age or race | X | X |
| --- | --- | --- |

**Table S2.** Association between natural food folate intake and blood folate concentration among females aged 12–49 years, based on 31 studies identified in the systematic review process 1.

| Reference | Population characteristics 1 | Dietary assessment method | Biomarker | Blood assay method 1 | Timing of exposure *Exposure level* | Natural food folate intake (µg DFE/day) | Serum/Plasma folate (nmol/L) | RBC folate  (nmol/L) |
| --- | --- | --- | --- | --- | --- | --- | --- | --- |
| Control trial | | | | | | | | |
| Abratte, C.  2008 [7] | 43 female Age: 25 (18–44) 1 *Staff and students from Cal Poly Pomona University and nearby community* Mexican American **Pomona, CA, USA**  1/2000–12/2001 | **Baseline:**  Unavailable  **Intervention:**  Folate feeding study  (14 week)  *Food folate measured with microbiologic trienzyme extraction* | Serum folate | *Microbiologic assay* *L. casei* | **Baseline: week 0** ^5^  **Depletion: week 7** *135 μg DFE/day*  **Repletion: week 14** 2  *400 μg DFE/day*  *800 μg DFE/day* | N/A  ~135  N/A | N/A  12.9 ± 0.7 2  N/A | N/A  Unavailable  N/A |
| Hung, J. 1  2006 [8] | 32 female Age: 22 (19–46) 1 *Staff and students from Cal Poly Pomona University and nearby community* African American, Arabian, Asian, Caucasian, Mexican American **Pomona, CA, USA**  1/2002–4/2003 | **Baseline:**  Unavailable  **Intervention:**  Natural food folate feeding study  (14 week)  *Food folate measured with ESHA Food Processor Nutrient Data Base (version 7.81; ESHA Research)* | Serum folate RBC folate | *Microbiologic assay L. casei* | **Baseline: week 0** ^5^  **Restriction: week 2** *135 μg DFE/day*  **Treatment: week 12–14** 1 *400 μg DFE/day*  *800 μg DFE/day* | N/A  133 ± 18 1  426 ± 44 10  835 ± 73 10 | N/A    19.3 ± 1.4 ^6^  18.4 ± 9.1 10  29.4 ± 12.9 10 | N/A  1430 ± 63 ^6^    1160 ± 285 10  1554 ± 326 10 |
| Perry, C.  2004 [9] | 42 female Age: 24.6 (19–44) ^4^ *Staff and students from Cal Poly Pomona University and nearby community* African American, Caucasian, Mexican American  **Pomona, CA, USA**  1/2000–12/2002 | **Baseline:**  Unavailable  **Intervention:**  Folate feeding study  (14 week)  *Food folate measured with microbiologic trienzyme extraction* | Serum folate RBC folate | *Microbiologic assay* *L. casei* | **Baseline: week 0** ^5^  **Folate depletion: week ^7^** *135 μg DFE/day*  **Folate repletion: week 14** ***^5^***  *400 μg DFE/day*  *800 μg DFE/day* | N/A  135 ± 9 ^6^  N/A | N/A  13.6 ± 1.1 ^6^  N/A | N/A  1117 ± 66 ^6^  N/A |
| Shelnutt, K. 2003 [10] | 41 female Age: 20–30 1  *Recruitment method unclear* Non-Hispanic black, non-Hispanic white **Gainesville, FL, USA**  2001 | **Baseline:**  Unavailable  **Intervention:**  Folate feeding study  (14 week)  *Food folate measured with microbiologic trienzyme extraction* | Serum folate RBC folate | *Microbiologic assay* *L. casei* | **Baseline: week 0** ^5^  **Depletion: week 7** *115 μg DFE/day*  **Repletion: week 14** ^5^  *400 μg DFE/day* | N/A    115 ± 20 10  N/A | N/A    17.6 ± 6.7 10  N/A | N/A    1336 ± 354 10  N/A |
| Wright, A. ^7^ 2010 [11] | 64 male  99 female (15 1) Age: 18–65 11 *Volunteers; from the Institute of Food Research's Human Nutrition Unit Database and community* **United Kingdom** | **Baseline:**  Weighed food record  (7 days)  **Intervention:**  Weighed food record  (7 days)  *Food folate measured using Diet Cruncher for Windows (WayDownSouth Software)* | Plasma folate RBC folate | *Microbiologic assay* *L. casei* | **Baseline: week 0**  **Intervention: week 16** *Additional 200* *μg DFE/day* | 274.1 ± 29.0 6    405.2 ± 31.9 ^6^ | 22.9 ± 4.1^6^  27.4 ± 3.7 ^6^ | 797.8 ± 106.8 ^6^  896.4 ± 135.0 ^6^ |
| Cohort | | | | | | | | |
| Dominguez-Salas, P.  2013 [12] | 62 female (28–30 for intake, 20–29 for blood)  Age: 31 (18–45) ^4^  *Random sample of women of reproductive age in three villages*  **West Kiang, Gambia**  7/2009–6/2010 | 24 h recall  (2 days × 12 months)  *Food folate measured by a surface plasmon resonance inhibition assay (Biacore Q assay)* | Plasma folate | Ion capture assay  AxSyM analyzer  Abbot Laboratories | 12 months  Rainy season  Dry season | 131.7 (69.2-251.5) 1  120.5 (112.3, 129.2) 13  138.4 (129.0, 148.6) 13 | 14.9 (6.6-33.4) 13  13.4 (12.2, 14.9) 13  12.5 (11.4, 13.9) 13 | Unavailable  Unavailable  Unavailable |
| Yang, Q.  2012 [13] ^7^ | 33,994 male and female (Phase 1: 2195 for intake, 2079 for blood; Phase 2: 2650 for intake, 2610 for blood 12)  Age: 2 months+  *Nationally representative sample of the US population*  **USA**  Phase 1: 10/88–10/91  Phase 2: 10/92–10/94 | 24 h recall (1 day) | Serum folate | *Radio competitive protein binding assay*  Quantaphase II  Bio-Rad Laboratories | Phase 1  Phase 2 | 170.69 (3.844) 1  175.32 (3.713) 14 | 9.04 (0.16) ^14^,15  10.42 (0.41) ^14^,15 | Unavailable  Unavailable |
| Cross-sectional | | | | | | | | |
| Chew, S. ^7^  2011 [14] | 42 male  58 female (58 12) Age, F: 20–45 11 *Random sample of staff and postgrad students at Universiti Putra Malaysia* Chinese, Malay  **Serdang, Selangor, Malaysia** | 24 h recall (1 day) | Serum folate RBC folate | *Microbiologic assay L. casei* | N/A | 321.934 ± 67.747 10 | 10.289 ± 4.293 10 | 219.046 ± 36.557 10 |
| Jang, H.  2013 [15] | 79 male  90 female (75 12)  Age: 20.9 ± 1.6 10  *College students recruited from a Chungbuk National University internet ad*  Korean  **Cheongju, Korea**  4/2009 | 24 h recall (3 days) | Serum folate  RBC folate | *Microbiologic assay L. casei* | N/A | 328.7 ± 119.2 10 | 27.6 ± 13.4 ^1,^10 | 906.2 ± 220.9 10^,^15 |
| Khor, G. ^7^  2006 [16] | 399 female (349 12)  Age: 23 (18–40) ^8^  *Recruited from universities and other suburban work sites* Chinese, Indian, Malay  **Kuala Lumpur, Malaysia**  1/2005–3/2005 | 24 h recall (1 day) | Plasma folate  RBC folate | *Microbiologic assay*  *L. casei* | N/A | **Total**  222.7 ± 130.7 10  **Malay**  224.4 ± 136.8 10  **Chinese**  212.6 ± 131.8 10  **Indian**  231.2 ± 122.9 10 | 13.13 ± 8.14 10  10.77 ± 5.29 10  16.29 ± 9.14 10  12.51 ± 8.67 10 | 662.1 ± 286.5 10  613.3 ± 210.4 10  683.3 ± 291.9 10  694.9 ± 343.6 10 |
| Han, Y.  2005 [17] | 44 male  62 female (62 12) Age, F: 20.4 ± 1.6 10 *Volunteers; students from Chungbuk National University* Korean **Cheonju,** **Korea** 3/1999 | 24 h recall (3 days) | Serum folate RBC folate | *Microbiologic assay* *L. casei* | N/A | 305 ± 119 10 | 17.1 ± 6.8 10 | 772 ± 240 10 |
| Kim, H.  2008 [18] | 36 female Age: 23.8 ± 5.8 10 *Volunteers from two cities* Korean **Gwangju and Sunchon, Korea** | Weighed food record  (3 days) | Plasma folate RBC folate | *Microbiologic assay* *L. casei* | N/A | 206.9 ± 90.8 10 | 23.8 ± 8.4 10^,^15 | 566.3 ± 176.3 10^,^15 |
| Kwanbunjan, K.  2008 [4] | 136 female Age: 15–45 11 *Housewives in each household*  Thai **Chaloem Phra Kiat, Nan, Thailand** | 24 h recall (1 day) | Serum folate RBC folate | *Microbiologic assay* *L. casei* | N/A | **15**–**29 years**  74.2 ± 40.8 10  **30**–**45 years**  80.6 ± 47.8 10 | 14.0 ± 9.1 10^,^15  19.3 ± 12.5 10^,^15 | 598.9 ± 199.9 10^,^15  455.2 ± 237.3 10^,^15 |
| Costa de Carvalho, M.  1996 [19] | 157 male  180 female (112 12) Age, F: 43.3 ± 10.9 10  *Subjects recruited at health examination center* **Dijon, France** 11/1985–11/1986 | Food record  (7 days) | Serum folate | *Radio competitive protein binding assay*  Immunophase  Corning Medical and Scientific | N/A | **18–29 years**  267 ± 53 10  **30–39 years**  274 ± 70 10  **40–49 years**  270 ± 69 10 | 16.3 ± 5.6 10  17.2 ± 5.4 10  18.0 ± 6.2 10 | Unavailable  Unavailable  Unavailable |
| Glew, R. ^7^  2001 [20] | 42 male  79 female (69 12)  Age, F: 32.0 ± 13.0 10  *Population based sample of adults*  Fulani  **Jos Plateau, Nigeria**  7/1999–8/1999 | Recall (7 days)  FFQ | Serum folate | *Radio competitive protein binding assay*  Immuno 1  Technicon | N/A | 124.9 ± 15.0 ^1,^10^,^ | 8.8 ± 7.9 10 | Unavailable |
| Haddad, E. ^7^  1999 [21] | 20 male  25 female (17 12) Age: 20–60 11 *Volunteers; Loma Linda University or health-care facilit*y *employees* **Loma Linda, CA, USA** 1993 | 24 h recall (4 days) | Serum folate | *Radio competitive protein binding assay*  Quantaphase II  Bio-Rad Laboratories | N/A | 383 ± 175 10 | 31.0 ± 13.1 10 | Unavailable |
| Liu, J. ^7^  2013 [22] | 121,700 for intake; 32,826 for blood; female  (3279 for intake; 77 for blood 12)  Age: 30–55 11  *Participants from the Nurses’ Health Study*  **USA**  1989–1990 | FFQ | Plasma folate | *Radio competitive protein binding assay*  Quantaphase II  Bio-Rad Laboratories | 1990 | 253.6 (106.5) 10 | 20.3 (57.2) 10 | Unavailable |
| Pathak, P.  2004 [5] | 288 female Age: 20.3 ± 2.2 10 *Population-based sample of married women*  Indian **Ballabhgarh, District Faridabad, Haryana, India** 11/2000–10/2001 | 24 h recall (1 day) | Serum folate | *Radio competitive protein binding assay* | N/A | 49.2 ± 20.1 10 | 11.8 ± 7.7 10^,^15 | Unavailable |
| Rasmussen,  L. ^7^  2000 [23] | 578 female (4512) Age: 25–6511 *Participants randomly selected from the civil registration system* **Copenhagen and Aalborg, Denmark** 3/1998- 6/1998 | Food record  (4 days) | RBC folate | *Radio competitive protein binding assay*  ICN Pharmaceuticals | N/A | 257 ± 74 10;  263 (200–296) 2 | Unavailable | 637 ± 235 10;  583 (456–770) 17 |
| Sofi, F. ^7^  2006 [24] | 367 male  565 female (273 12) Age, F: 46 (21–78) ^8^ *Randomly selected subjects from population registers* **Florence, Italy** 1/2002–1/2004 | FFQ | Serum folate | *Radio competitive protein binding assay*  ICN Diagnostic Product Corporation | N/A | 348.3 ± 122.3 10 | 12.7 ± 6.1 10^,^ 15 | Unavailable |
| Vandevijvere, S.  2013 [25] | 374 female  Age: 14.7 (1.2) 10  *Adolescents recruited from school sites*  **Vienna (Austria), Gent (Belgium), Lille (France), Dortmund (Germany), Athens, Heraklion (Greece), Pecs (Hungary), Rome (Italy), Zaragoza (Spain), Stockholm (Sweden)** | 24 h recall (2 days)  FFQ | Plasma folate  RBC folate | Competitive immunoassay  Immulite 2000  DPC Biermann GmbH | N/A | 180 (85) 10 | 18.2(9.6) 10 | 761.6(299.7) 10 |
| Agodi, A. ^7^  2014 [26] | 476 female (223 for intake; 121 for blood 12)  Age: 28.4 (14–49) 4;  27 (23–33) 17  *Healthy women referred to laboratory of the S. Bambino Hospital for preconceptional, prenatal and post-partum care*  **Catania, Italy**  7/2008-3/2011 | FFQ | RBC folate | *Chemiluminescent competitive protein binding assay*  Elecys Folate III  Roche Diagnostics | N/A | 220.6 (116.7) 10  202.8 (47.4-773.0) ^8^ | Unavailable | 270.9 (88.5) 10  250.8  (114.4–542.5) ^8^ |
| Castetbon,  K. ^7^  2009 [27] | 1126 male  1989 female (615 12)  Age: 18–74 11  *Randomly selected sample of non-institutionalized mainland population*  **France**  2/2006–3/2007 | 24 h recall (3 days) | Plasma folate | *Chemiluminescent competitive binding protein assay* | N/A | 285.5 (274.8–296.3) 2 | 14.5 (13.6–15.4) 15^,^18 | Unavailable |
| Henriquez,  P. ^7^  2004 [28] | 257 male  344 female (188 12) Age: 18–75 11 *Representative sample from each of 7 islands* **Canary Islands, Spain** 1996–1998 | FFQ | Serum folate RBC folate | *Microparticle capture chemoluminescence assay*  AXSYM Folate  Abbott Laboratories | N/A | 280.87 ± 147.74 10 | 17.9 ± 5.8 10^,^15 | 473 ± 173 10^,^15 |
| Hiraoka, M. 2001 [29] | 150 female Age: 21–22 11 *Students from Kagawa Nutrition University*  Japanese **Saitama, Japan** 4/1996, 4/1997 | Weighed food record  (3 days) | Serum folate | *Chemiluminescent competitive binding protein assay*  ACS:180  Bayer Diagnostics | N/A | 184 ± 75 10 | 17.9 ± 5.2 10 | Unavailable |
| Hiraoka, M. 2004 [30] | 340 female Age: 20–22 11 *Students from Kagawa Nutrition University* Japanese **Saitama, Japan** | Weighed food record  (3 days) | Serum folate | *Chemiluminescent competitive binding protein assay*  ACS Folate II  ADVIA Centaur  Bayer Diagnostics | N/A | 323 ± 133 10 | 17.2 (12.5–23.6) 2 | Unavailable |
| Al Khatib, L.  2006 [31] | 470 female Age: 28.6 ± 8.710 *Random sample of women attending one of 23 randomly selected health centers*  **Lebanon** 5/2003–12/2003 | 24h recall (1 day) | Plasma folate | *Microparticle capture chemoluminescence assay*  AXSYM Folate Abbott Laboratories | N/A | 275.8 ± 216.2 10 | 19.0 ± 6.1 10^,^15 | Unavailable |
| Kondo, A. ^7^ *Unpublished data* [32] | 72 female (68 12) Age: 20.2 ± 1.6 10;  (19–28) 11 *Volunteers; students from the Kasugai-Komaki College for Nurse Education* **Aichi Prefecture, Japan** 2005 | Food record  (3 days) | Plasma folate | *Chemiluminescent competitive binding protein assay* | N/A | 299.7 ± 392.1 10 | 16.3 ± 6.6 10^,^15;  (9.1-54.4) 11^,^15 | Unavailable |
| Lee, Y.  2011 [33] | 116 female Age: 18–44 11 *Volunteers; Buddhist vegetarian nuns, Catholic omnivore nuns, college student omnivores* Korean **Daegu and Gyeongbuk Provinces, South Korea** 10/2002–3/2003 | Food record  (3 days) | Serum folate | *Chemiluminescent competitive binding protein assay*  Elecsys 2010  Roche Diagnostics | N/A | **Buddhist vegetarian nuns**  340 (296–430) 17  **Catholic omnivore nuns**  267 (241–347) 17  **College student omnivores**  177 (152–219) 17 | 22.9 (18.6–29.5) 17  19.7 (17.9–24.9) 17  15.6 (11.3–17.9) 17 | Unavailable  Unavailable  Unavailable |
| Planells, E. ^7^  2003 [34] | 1813 male  1715 female (1148 for intake, 101 for blood 12) Age: 25–60 11 *Probablistic, stratified, random sample of resident adults* **Andalusia, Spain** | 24 h recall (2 days) | Plasma folate | *Enzyme competitive protein binding assay*  Ridascreen Folsaure  r-biopharm GmbH | N/A | 195.0 ± 109.4 10 | 16.7 ± 8.5 10^,^15 | Unavailable |
| Watanabe,  H. ^7^  2012 [35] | 141 female (122 12)  Age: 20.0 ± 2.1 10  *Participants recruited from 2 universities and a technical school*  **Yamanashi and Shiga Prefectures, Japan**  10/2009–11/2009 | BDHQ | Serum folate | *Chemiluminescent competitive protein binding assay*  ACS Folate II  Siemens Healthcare Diagnostics | N/A | 231.20 (72.84–774.46) ^8^; 256.26 (133.47) 10 | 18.74 (4.53–45.32) ^8^; 19.69 (9.34) 10 | Unavailable |

^1^ CA, California; DFE, dietary folate equivalent; FFQ, food frequency questionnaire; FL, Florida; *L.* *casei, Lactobacillus* *casei*; N/A, not applicable; RBC, red blood cell; USA, United States of America; BDHQ, brief, self-administered, diet-history questionnaire; ^2^ As presented in the original paper, unless otherwise noted: sample size; age (F denotes female only if given); sampling description; ethnicity/nationality (if given); study location; data collection periods (if given); ^3^ Blood folate assay characteristics: assay method; organism (if relevant); assay name; manufacturer; ^4^ Mean (range); 5 This phase of the study included consumption of folic acid and is therefore not presented; 6 Mean ± SEM; 7 Author contacted for blood folate concentration or folate intake data for
non-pregnant, non-lactating women 12–49 years of age. Additional data were provided by study author; 8 Median (range); 9 Average of blood and intake over treatment period; 1^0^ Mean ± SD; 1^1^ Range; 1^2^ Denotes sample size for the subpopulation of women aged 12–49 in this study. All blood folate concentrations and total folate intake are specific to subpopulation; 1^3^ Geometric mean (95% CI); 1^4^ Geometric mean (SE); 1^5^ Data reported in ng/mL converted to nmol/L using: 1 ng/mL = 2.266 nmol/L; 1^6^ Nutrient analysis was carried out using the 7-day dietary recall and applicable data from the FFQ; 1^7^ Median (IQR);
1^8^ Mean (95% CI); 1^9^ Geometric mean (range).

**Table S3.** Association between natural food folate intake and blood folate concentration in populations of >50% female and median/mean age 12–49 years, based on five studies identified in the systematic review process ^1^.

| Reference | Population characteristics 1 | Dietary assessment method | Biomarker | Blood assay method 1 | Timing of exposure *Exposure level* | Natural food folate intake  (µg DFE/day) | | | Serum/Plasma folate  (nmol/L) | RBC folate (nmol/L) |
| --- | --- | --- | --- | --- | --- | --- | --- | --- | --- | --- |
| Control trial | | | | | | | | | | |
| Brouwer, I.  1999 [36] | 25 male  42 female Age: 18–45 1 *Volunteers* **Netherlands** | FFQ  **Intervention:**  Folate feeding study  (4 week)  Calculated exposure*: food folate measured using the Dutch Nutrient Database*  Analyzed exposur*e: food folate measured by microbiologic assay* | Plasma folate RBC folate | *Microparticle enzyme competitive protein binding assay*  IMX analyzer  Abbott Laboratories | **Placebo**  *Calculated: 226 ± 9* 2  **Dietary folate** *Calculated: 594 ± 27* ^5^ | | Analyzed 3:  210 ± 49 ^5^  Analyzed ^6^:  560 ± 184 ^5^ | | Week 0:  13.2 ± 3.4 ^5^ Week 2:  12.9 ± 3.6 ^5^ Week 4:  12.7 ± 2.9 ^5^  Week 0:  13.8 ± 3.0 ^5^ Week 2:  20.1 ± 4.0 ^5^ Week 4:  20.4 ± 3.5 ^5^ | Week 0:  347 ± 79 ^5^  Week 2: Unavailable  Week 4:  345 ± 69 ^5^  Week 0:  338 ± 81 ^5^ Week 2: Unavailable  Week 4:  400.1 ± 114 ^5^ |
| Silaste, M.  2003 [37] | 37 female Age: 43 ± 10 ^5^  *Volunteers; University Hospital of Oulu staff* **Oulu, Finland** | **Baseline:** Food record  (4 days)  **Intervention:**  Folate feeding study (15 week)  *Food folate measured from identical food portions collected daily* | Serum folate RBC folate | *Radio competitive protein binding assay*  Quantaphase II  Bio-Rad Laboratories | **Baseline: week 2**  **Low-folate: week 7** *200 μg DFE/day*  **Washout: week 10** *Habitual diet*  **High-folate: week 15** *600 μg DFE/day* | | 284 ± 56 ^5^  221 ± 24 ^5^  Unavailable  596 ± 66 ^5^ | | 11.0 ± 3.7 ^5^  11.0 ± 3.0 ^5^  13.3 ± 5.5 ^5^  19.3 ± 6.4 ^5^ | 389 ± 122 ^5^  412 ± 120 ^5^  Unavailable  464 ± 138 ^5^ |
| Cross-sectional | | | | | | | | | | |
| Mennen, L.  2002 [38] | 931 male  1139 female (310 F with dietary intake data) Age, F: 46.8 ± 6.5 ^5^; (35–60) ^4^ *Random sample of subjects from the Supplementation with Antioxidant Vitamins and Minerals Study* **France** 1994 | 24 h recall (6 days) | RBC folate | *Microbiologic assay* | N/A | | | 268.2 ± 91.5 ^5^ | Unavailable | 606.1 ± 208.8 ^5^ |
| Nagata, C.  2003 [39] | 291 female Age: 42.5 ± 5.3 ^5^  *Participants in a health check-up program*  Japanese **Gifu, Japan** 9/1996–8/1997 | FFQ | Serum folate | *Chemiluminescent competitive binding protein assay*  ACS:180  Bayer Diagnostics | N/A | | | 520.2 ± 288.6 ^5^ | 11.8 ± 3.9 ^5^ | Unavailable |
| Taguchi, T.  2012 [40] | 192 female  Age: 15.4 ± 0.1 ^5^;  (12–18) ^4^  *Junior and senior high school students*  Japanese  **Hyogo Prefecture, Japan**  6/2009 | FFQ | Serum folate | Unknown | N/A | | | 239 ± 7.1 ^5^ | 13.8 ± 0.5 ^5^^,^6;  (5.4–45.8) ^4,7^ | Unavailable |

1 DFE, dietary folate equivalent; FFQ, food frequency questionnaire; *L*. *casei,, Lactobacillus* *casei*; N/A, not applicable; RBC, red blood cell; 2 As presented in the original paper, unless otherwise noted: sample size; age (F denotes female only if given); sampling description; ethnicity/nationality (if given); study location; data collection periods (if given); 3 Blood folate assay characteristics: assay method; organism (if relevant); assay name; manufacturer; 4 Range;
5 Mean ± SD; 6 Duplicate portions of the diet were collected for a fictitious participant and the folate content was analyzed in a subsample by microbiological assay with *Lactobacillus casei*; 7 Data reported in ng/mL converted to nmol/L using: 1 ng/mL = 2.266 nmol/L.

**Table S4**. Summary risk of bias tables for randomized controlled trials using Cochrane tool ^1^.

|  | | Selection bias | | | Performance bias | Detection bias | | | Attrition bias | | Reporting bias | Overall Risk of Bias ^2^ |
| --- | --- | --- | --- | --- | --- | --- | --- | --- | --- | --- | --- | --- |
| *Study design* | **Domains**  **Papers** | Sequence generation^2^ | Allocation concealment^2^ | Uniform inclusion/exclusion criteria^3^ | Blinding participants/personnel | Blinding outcome | Valid & reliable exposure assessment ^3^ | Valid & reliable outcome assessment ^3^ | Incomplete outcome data | Appropriate follow-up time | Selective reporting |  |
| *RCT* | *Brouwer, I. ^4^, 1999* | H | H | L | M | L | L | M ^5,6^ | L | L ^5^/H ^6^ | L | **H** ^5,6^ |
|  | *Silaste, M., 2003* | U | U | L | M | L | L | M ^5^/H ^6^ | L | L ^5^/H ^6^ | L | **M** ^5^**/H** ^6^ |
|  | *Perry, C., 2004* | U | U | L | - ^7^ | L | L | L ^5,6^ | L | L ^5^/H ^6^ | L | **L** ^5^**/H** ^6^ |
|  | *Hung, J., 2006* | U | U | L | M | L | L | L ^5,6^ | L | L ^5^/H ^6^ | L | **M** ^5^**/H** ^6^ |
|  | *Abratte, C., 2008* | U | U | L | - ^7^ | L | L | L ^5^ | L | L ^5^ | L | **L** ^5^ |
|  | *Wright, A., 2010* | U | U | L | - ^7^ | L | L | L ^5,6^ | L | L ^5^/H ^6^ | L | **L** ^5^**/H** ^6^ |

^1^ Risk of bias assessment: L = low; M = moderate; H = high; U = unclear; ^2^ Because the majority of randomized controlled trials did not provide study details for sequence generation and allocation concealment, these domains were not included in the assessment of overall risk of bias for each study and were categorized as unclear; ^3^ Cochrane recommends stating potential sources of bias not addressed in the other domains of the risk of bias tool. Additional potential sources of bias identified in our systematic review include uniform inclusion/exclusion criteria across comparison groups, and valid & reliable assessments for exposure and outcome and appropriate follow-up time; ^4^ Study was not a randomized controlled trial but was a controlled trial with multiple intervention arms and thus the Cochrane tool was used to assess risk of bias; ^5^ Plasma or serum folate; ^6^ Red blood cell folate; ^7^ Potential bias from the blinding of study participants and personnel would not have affected studies in which only a subset of the data (i.e. blood folate measurements at week 7 depletion time point or measurements from only one intervention group) were used in the review and thus were not given a risk of bias score for this domain.

**Table S5**. Summary risk of bias tables for non-randomized controlled trials and observational studies using adapted RTI
item bank ^1^.

|  | | Selection bias | | Detection bias | | | Attrition bias | | Confounding | Overall Risk of Bias |
| --- | --- | --- | --- | --- | --- | --- | --- | --- | --- | --- |
| *Study design* | **Domains**  **Paper** | Clear inclusion/ exclusion criteria | Appropriate sample selection | Valid assessment of: | | | Incomplete outcome data | Appropriate follow-up time | Confounder adjustments, stratification |  |
|  |  |  |  | inclusion/ exclusion | exposure | outcome |  |  |  |  |
| *Non-randomized controlled trial* | *Shelnutt, K., 2003* | L | M | M | L | L ^2,3^ | - ^4^ | L ^2^**/**H ^3^ | M | **M** ^2^/**H** ^3^ |
| *Cohort* | *Yang, Q., 2012* | L | L | L | M | M ^2^ | - ^4^ | - ^5^ | H | **H** ^2^ |
|  | *Domingues-Salas, P., 2013* | L | L | L | M | M ^2^ | - ^4^ | L ^2^ | M | **M** ^2^ |

**Table S5.** *Cont.*

| *Cross-sectional* | *Costa de Carvalho, M., 1996* | M | M | M | M | M ^2^ | - | - | M | M ^2^ |
| --- | --- | --- | --- | --- | --- | --- | --- | --- | --- | --- |
|  | *Haddad, E., 1999* | L | M | M | M | M ^2^ | - | - | H | **H** ^2^ |
|  | *Rasmussen, L., 2000* | L | M | L | M | M ^3^ | - | - | M | **M** ^3^ |
|  | *Glew, R., 2001* | M | M | M | M | M ^2^ | - | - | M | **M** ^2^ |
|  | *Hiraoka, M., 2001* | M | M | M | M | M ^2^ | - | - | L | **M** ^2^ |
|  | *Mennen, L., 2002* | L | M | M | M | L ^3^ | - | - | M | **M** ^3^ |
|  | *Nagata, C., 2003* | L | M | M | H | M ^2^ | - | - | L | **H** ^2^ |
|  | *Planells, E., 2003* | L | L | M | M | M ^2^ | - | - | M | **M** ^2^ |
|  | *Henriquez, P., 2004* | L | L | L | H | M ^2,3^ | - | - | M | **H** ^2,3^ |
|  | *Hiraoka, M., 2004* | L | M | M | M | M ^2^ | - | - | L | **M** ^2^ |
|  | *Pathak, P., 2004* | L | M | M | M | M ^2^ | - | - | L | **M** ^2^ |
| *Cross-sectional* | *Han, Y., 2005* | M | M | M | M | L ^2,3^ | - | - | L | M ^2,3^ |
|  | *Khatib, L., 2006* | L | M | M | M | M^2^ | - | - | M | **M** ^2^ |
|  | *Kondo, A., 2005* | H | M | M | M | M ^2^ | - | - | L | **H** ^2^ |
|  | *Khor, G., 2006* | L | M | M | M | L ^2,3^ | - | - | L | **M** ^2,3^ |
|  | *Sofi, F., 2006* | M | L | L | H | M ^2^ | - | - | M | **H** ^2^ |
|  | *Kim, H., 2008* | M | M | M | M | L ^2,3^ | - | - | M | **M** ^2,3^ |
|  | *Kwanbujan, K., 2008* | L | M | M | M | L ^2,3^ | - | - | L | **M** ^2,3^ |
|  | *Castetbon, K., 2009* | L | L | L | M | M ^2^ | - | - | M | **M** ^2^ |
|  | *Chew, S., 2011* | L | M | M | M | L ^2,3^ | - | - | H | **H** ^2,3^ |
|  | *Lee, Y., 2011* | L | M | M | M | M ^2^ | - | - | L | **M** ^2^ |
|  | *Taguchi, T., 2012* | M | M | L | H | H ^2^ | - | - | L | **H** ^2^ |
|  | *Watanabe, H., 2012* | L | M | M | H | M ^2^ | - | - | L | **H** ^2^ |
|  | *Jang, H., 2013* | M | M | M | M | L ^2,3^ | - | - | L | **M** ^2,3^ |
|  | *Liu, J., 2013* | L | M | M | H | M ^2^ | - | - | M | **H** ^2^ |
|  | *Agodi, A., 2014* | L | M | L | H | M ^3^ | - | - | L | **H** ^3^ |
|  | *Vandevijvere, S., 2013* | L | L | L | M | L ^2^M ^3^ | - | - | L | **M** ^2,3^ |

^1^ Risk of Bias assessment: L = low; M = moderate; H = high; ^2^ Plasma or serum folate; ^3^ Red blood cell folate; ^4^ Not applicable because studies did not have multiple comparison groups; ^5^ Not applicable because study used only cross-sectional data.

**References**

1. Higgins, J.P.T.; Altman, D.G.; Sterne, J.A.C. Chapter 8: Assessing risk for bias in included studies.
   In *Cochrane Handbook for Systematic Reviews of Interventions 510*; Higgins, J., Green, S., Eds.;
   The Cochrane Collaboration: Oxford, UK, 2011.
2. Viswanathan, M.; Berkman, N.D. Development of the RTI item bank on risk of bias and precision of observational studies. *J. Clin. Epidemiol.* **2012**, *65*, 163–178, doi:10.1016/j.jclinepi.2011.05.008.
3. Souverein, O.W.; Dullemeijer, C.; van’t Veer, P.; van der Voet, H. Transformations of summary statistics as input in meta-analysis for linear dose-response models on a logarithmic scale:
   A methodology developed within EURRECA. *BMC Med. Res. Methodol.* **2012**, *12*,
   doi:10.1186/1471-2288-12-57.
4. Kwanbunjan, K.; Thepouyporn, A.; Songmuaeng, K.; Nakosiri, W.; Cheeramakara, C.; Chusongsang, Y.; Laisupasin, P.; Tunsakul, S.; Chantaranipapong, Y.; Pooudong, S.; *et al.* Food behavior and folate status of hill-tribe schoolchildren and women of childbearing age on the northern border of Thailand. *Southeast Asian J. Trop. Med. Public Health* **2008**, *39*, 353–361.
5. Pathak, P.; Saxena, R.; Kapoor, S.K.; Dwivedi, S.N.; Singh, R.; Kapil, U. Status of serum ferritin and folate levels amongst young women in a rural community of Haryana, India. *Nepal Med. Coll. J.* **2004**, *6*, 13–16.
6. Willett, W. *Nutritional Epidemiology*, 2nd ed.; Oxford University Press: New York, NY, USA, 1998.
7. Abratte, C.M.; Wang, W.; Li, R. Moriarty DJ, Caudill MA. Folate intake and the MTHFR C677T genotype influence choline status in young Mexican American women. *J. Nutr. Biochem.* **2008**, *19*, 158–165, doi:10.1016/j.jnutbio.2007.02.004.
8. Hung, J.; Yang, T.L.; Urrutia, T.F.; Li, R. Perry, C.A.; Hata, H.; Cogger, E.A.; Moriarty, D.J.; Caudill, M.A. Additional food folate derived exclusively from natural sources improves folate status in young women with the MTHFR 677 CC or TT genotype. *J. Nutr. Biochem.* **2006**, *17*, 728–734, doi:10.1016/j.jnutbio.2005.11.009.
9. Perry, C.A.; Renna, S.A.; Khitun, E.; Ortiz, M.; Moriarty, D.J.; Caudill, M.A. Ethnicity and race influence the folate status response to controlled folate intakes in young women. *J. Nutr.* **2004**, *134*, 1786–1792.
10. Shelnutt, K.P.; Kauwell, G.P.; Chapman, C.M.; Gregory, J.F., 3rd; Maneval, D.R.; Browdy, A.A.; Theriaque, D.W.; Bailey, L.B. Folate status response to controlled folate intake is affected by the methylenetetrahydrofolate reductase 677C-->T polymorphism in young women. *J. Nutr.* **2003**, *133*, 4107–4111.
11. Wright, A.J.; King, M.J.; Wolfe, C.A.; Powers, H.J.; Finglas, P.M. Comparison of
    (6 S)-5-methyltetrahydrofolic acid *vs.* folic acid as the reference folate in longer-term human dietary intervention studies assessing the relative bioavailability of natural food folates: comparative changes in folate status following a 16-week placebo-controlled study in healthy adults. *Br. J. Nutr.* **2010**, *103*, 724–729, doi:10.1017/s0007114509992339.
12. Dominguez-Salas, P.; Moore, S.E.; Cole, D.; da Costa, K.A.; Cox, S.E.; Dyer, R.A.; Fulford, A.J.; Innis, S.M.; Waterland, R.A.; Zeisel, S.H.; *et al.* DNA methylation potential: dietary intake and blood concentrations of one-carbon metabolites and cofactors in rural African women. *Am. J. Clin. Nutr.* **2013**, *97*, 1217–1227, doi:10.3945/ajcn.112.048462.
13. Yang, Q.; Bailey, L.; Clarke, R.; Flanders, W.D.; Liu, T.; Yesupriya, A.; Khoury, M.J.; Friedman, J.M. Prospective study of methylenetetrahydrofolate reductase (MTHFR) variant C677T and risk of
    all-cause and cardiovascular disease mortality among 6000 US adults. *Am. J. Clin. Nutr.* **2012**, *95*, 1245–1253, doi:10.3945/ajcn.111.022384.
14. Chew, S.C.; Khor, G.L.; Loh, S.P. Association between dietary folate intake and blood status of folate and homocysteine in Malaysian adults. *J. Nutr. Sci. Vitaminol. (Tokyo)* **2011**, *57*, 150–155.
15. Jang, H.B.; Han, Y.H.; Piyathilake, C.J.; Kim, H.; Hyun, T. Intake and blood concentrations of folate and their association with health-related behaviors in Korean college students. *Nutr. Res. Pract.* **2013**, *7*, 216–223, doi:10.4162/nrp.2013.7.3.216.
16. Khor, G.L.; Duraisamy, G.; Peng Loh, S.; Green, T.J.; Skeaff, C.M. Dietary and blood folate status of Malaysian women of childbearing age. *Asia Pac. J. Clin. Nutr.* **2006**, *15*, 341–349.
17. Han, Y.H.; Yon, M.; Hyun, T.H. Folate intake estimated with an updated database and its association to blood folate and homocysteine in Korean college students. *Eur. J. Clin. Nutr.* **2005**, *59*, 246–254, doi:10.1038/sj.ejcn.1602065.
18. Kim, H.A.; Lim, H.S. Dietary folate intake, blood folate status, and urinary folate catabolite excretion in Korean women of childbearing age. *J. Nutr. Sci. Vitaminol. (Tokyo)* **2008**, *54*, 291–297.
19. De Carvalho, M.J.; Guilland, J.C.; Moreau, D.; Boggio, V.; Fuchs, F. Vitamin status of healthy subjects in Burgundy (France). *Ann. Nutr. Metab.* **1996**, *40*, 24–51.
20. Glew, R.H.; Williams, M.; Conn, C.A.; Cadena, S.M.; Crossey, M.; Okolo, S.N.; VanderJagt, D.J. Cardiovascular disease risk factors and diet of Fulani pastoralists of northern Nigeria. *Am. J. Clin. Nutr.* **2001**, *74*, 730–736.
21. Haddad, E.H.; Berk, L.S.; Kettering, J.D.; Hubbard, R.W.; Peters, W.R. Dietary intake and biochemical, hematologic, and immune status of vegans compared with nonvegetarians. Am *J. Clin. Nutr.* **1999**, *70*, 586S–593S.
22. Liu, J.J.; Prescott, J.; Giovannucci, E.; Hankinson, S.E.; Rosner, B.; De Vivo, I. One-carbon metabolism factors and leukocyte telomere length. *Am. J. Clin. Nutr.* **2013**, *97*, 794–799.
23. Rasmussen, L.B.; Ovesen, L.; Bulow, I.; Knudsen, N.; Laurberg, P.; Perrild, H. Folate intake, lifestyle factors, and homocysteine concentrations in younger and older women. Am *J. Clin. Nutr.* **2000**, *72*, 1156–1163.
24. Sofi, F.; Innocenti, G.; Dini, C.; Masi, L.; Battistini, N.C.; Brandi, M.L.; Rotella, C.M.; Gensini, G.F.; Abbate, R.; Surrenti, C.; *et al.* Low adherence of a clinically healthy Italian population to nutritional recommendations for primary prevention of chronic diseases. *Nutr. Metab. Cardiovasc. Dis*. **2006**, *16*, 436–444, doi:10.1016/j.numecd.2005.09.002.
25. Vandevijvere, S.; Geelen, A.; Gonzalez-Gross, M.; Van, T.V.P.; Dallongeville, J.; Mouratidou, T.; Dekkers, A.; Bornhorst, C.; Breidenassel, C.; Crispim, S.P.; *et al.* Evaluation of food and nutrient intake assessment using concentration biomarkers in European adolescents from the Healthy Lifestyle in Europe by Nutrition in Adolescence study. *Br. J. Nutr.* **2013**, *109*, 736–747.
26. Agodi, A.; Barchitta, M.; Quattrocchi, A.; Marchese, A.E.; Boffetta, P. Folate deficiency is not associated with increased mitochondrial genomic instability: Results from dietary intake and lymphocytic mtDNA 4977-bp deletion in healthy young women in Italy. *Mutagenesis* **2014**, *29*,
    101–106, doi:10.1093/mutage/get065.
27. Castetbon, K.; Vernay, M.; Malon, A.; Salanave, B.; Deschamps, V.; Roudier, C.; Oleko, A.; Szego, E.; Hercberg, S. Dietary intake, physical activity and nutritional status in adults: the French nutrition and health survey (ENNS, 2006–2007). *Br. J. Nutr.* **2009**, *102*, 733–743, doi:10.1017/S0007114509274745.
28. Henriquez, P.; Doreste, J.; Diaz-Cremades, J.; Lopez-Blanco, F.; Alvarez-Leon, E.;
    Serra-Majem, L. Folate status of adults living in the Canary Islands (Spain). *Int. J. Vitam. Nutr. Res.* **2004**, *74*, 187–192.
29. Hiraoka, M. Nutritional status of vitamin A, E, C, B1, B2, B6, nicotinic acid, B12, folate, and
    beta-carotene in young women. *J. Nutr. Sci. Vitaminol. (Tokyo)* **2001**, *47*, 20–27.
30. Hiraoka, M. Folate intake, serum folate, serum total homocysteine levels and methylenetetrahydrofolate reductase C677T polymorphism in young Japanese women. *J. Nutr. Sci. Vitaminol. (Tokyo)* **2004**, *50*, 238–245.
31. Al Khatib, L.; Obeid, O.; Sibai, A.M.; Batal, M.; Adra, N. Folate deficiency is associated with nutritional anaemia in Lebanese women of childbearing age. *Public Health Nutr.* **2006**, *9*, 921–927.
32. Kondo, A. (Tsushima Rehabilitation Hospital, Minami-Shinkai, Tsushima, Japan); Personal communication, 2012.
33. Lee, Y.; Krawinkel, M. The nutritional status of iron, folate, and vitamin B-12 of Buddhist vegetarians. *Asia Pac. J. Clin. Nutr.* **2011**, *20*, 42–49.
34. Planells, E.; Sanchez, C.; Montellano, M.A.; Mataix, J.; Llopis, J. Vitamins B6 and B12 and folate status in an adult Mediterranean population. *Eur. J. Clin. Nutr.* **2003**, *57*, 777–785.
35. Watanabe, H.; Ishida, S.; Konno, Y.; Matsumoto, M.; Nomachi, S.; Masaki, K.; Okayama, H.;
    Nagai, Y. Impact of dietary folate intake on depressive symptoms in young women of reproductive age. *J. Midwifery Womens Health* **2012**, *57*, 43–48.
36. Brouwer, I.A.; van Dusseldorp, M.; West, C.E.; Meyboom, S.; Thomas, C.M.; Duran, M.; van het Hof, K.H.; Eskes, T.K.; Hautvast. J.G.; Steegers-Theunissen, R.P. Dietary folate from vegetables and citrus fruit decreases plasma homocysteine concentrations in humans in a dietary controlled trial.
    *J. Nutr.* **1999**, *129*, 1135–1139.
37. Silaste, M.L.; Rantala, M.; Alfthan, G.; Aro, A.; Kesaniemi, A. Plasma homocysteine concentration is decreased by dietary intervention. *Br. J.Nutr.* **2003**, *89*, 295–301.
38. Mennen, L.I.; de Courcy, G.P.; Guilland, J.C.; Ducros, V.; Bertrais, S.; Nicolas, J.P.; Maurel, M.; Zarebska, M.; Favier, A.; Franchisseur, C.; *et al.* Homocysteine, cardiovascular disease risk factors, and habitual diet in the French Supplementation with Antioxidant Vitamins and Minerals Study.
    *Am. J. Clin. Nutr.* **2002**, *76*, 1279–1289.
39. Nagata, C.; Shimizu, H.; Takami, R.; Hayashi, M.; Takeda, N.; Yasuda, K. Soy product intake is inversely associated with serum homocysteine level in premenopausal Japanese women. *J. Nutr.* **2003**, *133*, 797–800.
40. Taguchi, T.; Mori, H.; Hamada, A.; Yamori, Y.; Mori, M. Serum folate, total homocysteine levels and methylenetetrahydrofolate reductase 677C>T polymorphism in young healthy female Japanese. *Asia Pac. J. Clin. Nutr.* **2012**, *21*, 291–295.

© 2015 by the authors; licensee MDPI, Basel, Switzerland. This article is an open access article distributed under the terms and conditions of the Creative Commons Attribution license (http://creativecommons.org/licenses/by/4.0/).
